# T2-Weighted 4D Magnetic Resonance Imaging for Application in Magnetic Resonance–Guided Radiotherapy Treatment Planning

**DOI:** 10.1097/RLI.0000000000000381

**Published:** 2017-04-28

**Authors:** Joshua N. Freedman, David J. Collins, Hannah Bainbridge, Christopher M. Rank, Simeon Nill, Marc Kachelrieß, Uwe Oelfke, Martin O. Leach, Andreas Wetscherek

**Affiliations:** From the *Joint Department of Physics, †CR UK Cancer Imaging Centre, and ‡Joint Department of Radiotherapy, The Institute of Cancer Research and The Royal Marsden NHS Foundation Trust, London, United Kingdom; and §Medical Physics in Radiology, German Cancer Research Center (DKFZ), Heidelberg, Germany.

**Keywords:** 4D MRI, motion model, motion vector field, MR-linac, radiotherapy treatment planning, 4D-T2w MRI

## Abstract

**Objectives:**

The aim of this study was to develop and verify a method to obtain good temporal resolution T2-weighted 4-dimensional (4D-T2w) magnetic resonance imaging (MRI) by using motion information from T1-weighted 4D (4D-T1w) MRI, to support treatment planning in MR-guided radiotherapy.

**Materials and Methods:**

Ten patients with primary non–small cell lung cancer were scanned at 1.5 T axially with a volumetric T2-weighted turbo spin echo sequence gated to exhalation and a volumetric T1-weighted stack-of-stars spoiled gradient echo sequence with golden angle spacing acquired in free breathing. From the latter, 20 respiratory phases were reconstructed using the recently developed 4D joint MoCo-HDTV algorithm based on the self-gating signal obtained from the k-space center. Motion vector fields describing the respiratory cycle were obtained by deformable image registration between the respiratory phases and projected onto the T2-weighted image volume. The resulting 4D-T2w volumes were verified against the 4D-T1w volumes: an edge-detection method was used to measure the diaphragm positions; the locations of anatomical landmarks delineated by a radiation oncologist were compared and normalized mutual information was calculated to evaluate volumetric image similarity.

**Results:**

High-resolution 4D-T2w MRI was obtained. Respiratory motion was preserved on calculated 4D-T2w MRI, with median diaphragm positions being consistent with less than 6.6 mm (2 voxels) for all patients and less than 3.3 mm (1 voxel) for 9 of 10 patients. Geometrical positions were coherent between 4D-T1w and 4D-T2w MRI as Euclidean distances between all corresponding anatomical landmarks agreed to within 7.6 mm (Euclidean distance of 2 voxels) and were below 3.8 mm (Euclidean distance of 1 voxel) for 355 of 470 pairs of anatomical landmarks. Volumetric image similarity was commensurate between 4D-T1w and 4D-T2w MRI, as mean percentage differences in normalized mutual information (calculated over all respiratory phases and patients), between corresponding respiratory phases of 4D-T1w and 4D-T2w MRI and the tie-phase of 4D-T1w and 3-dimensional T2w MRI, were consistent to 0.41% ± 0.37%. Four-dimensional T2w MRI displayed tumor extent, structure, and position more clearly than corresponding 4D-T1w MRI, especially when mobile tumor sites were adjacent to organs at risk.

**Conclusions:**

A methodology to obtain 4D-T2w MRI that retrospectively applies the motion information from 4D-T1w MRI to 3-dimensional T2w MRI was developed and verified. Four-dimensional T2w MRI can assist clinicians in delineating mobile lesions that are difficult to define on 4D-T1w MRI, because of poor tumor-tissue contrast.

## Abstract 2

Supplemental digital content is available in the text.

Designing a radiotherapy treatment plan (RTP) for lung and abdominal cancer is challenging due to the motion of the abdominal-thoracic region.^1^ An appropriate RTP should deliver the prescribed dose to the target and minimize dose to radiosensitive healthy structures. Unlike conventional free breathing scans where generic margins are added, 4-dimensional (4D) images provide information on individual tumor motion, which can be used to generate a more personalized RTP.^2^

Strategies have been proposed to manage abdominal-thoracic motion, such as breath-hold or gated treatment.^1^ Yet these approaches are suboptimal because they can be challenging for patients with primary lung malignancies or can lengthen treatment time. Motion information from image-guided radiotherapy (IGRT) is used to improve treatment delivery.^3^

Several imaging modalities can be used for IGRT, such as orthogonal kilovolt imaging, cone-beam computed tomography or, with the advent of hybrid MR-IGRT systems,^4–8^ magnetic resonance imaging (MRI). Compared with cone-beam computed tomography, MRI exhibits improved soft tissue contrast, is a nonionizing modality, and offers a range of clinically relevant contrasts.^9^ However, MRI is limited by intrinsic spatial uncertainty,^10^ and there is no direct relationship between electron density and the MRI signal, whereas a knowledge of electron density is required for dose calculations.^11^

In this manuscript, 4D MRI is defined as a time series of 3-dimensional (3D) MRI scan volumes, where each volume corresponds to a different time point in the respiratory cycle. In current clinical practice, 4D MRI could be used to inform an RTP by providing additional information, such as improved soft tissue contrast, absent in 4D CT.^9^ In the near future, 4D MRI might be acquired on hybrid MR-IGRT systems to provide information to support RTP adaptation with improved setup and beam guidance.^5^ Thus, there is a great need for robust methods for generating 4D MRI.

Previous work to produce 4D MRI can be loosely split into dynamic slice-selective 2-dimensional (2D) and volumetric 3D acquisition schemes. Two-dimensional MRI can be prospectively acquired at specific respiratory phases by triggering acquisition with the aid of a synchronized respiratory signal. A complete 4D MRI volume is then constructed by continuously measuring slices at all required planes and respiratory phases.^12^ However, triggering delays have been reported, where triggering of the current phase is ignored due to continuing measurement of the preceding phase, which can result in a long acquisition time.^13^

Alternatively, 4D MRI can be constructed retrospectively by sorting measured slices with the aid of a respiratory signal. Both external and internal respiratory signals have been utilized. Among others, the center of k-space (self-gating),^14,15^ body area,^16^ and diaphragm position^17–19^ have been used as internal respiratory signals. External respiratory signals are typically acquired using a breathing belt,^20–23^ but clock drifts or poor respiratory correlation have been reported.^18,24^ Most commonly, the amplitude or phase of the respiratory signal has been applied to sort slices into bins of respiratory phase. Retrospective schemes suffer from data incompleteness artifacts, where a slice was not acquired in all respiratory phases, but can be mitigated by prolonged image acquisition.^18,20^

Four-dimensional MRI constructed from aggregated and sorted 2D MRI often exhibits staircase artifacts, due to highly nonisotropic voxel sizes, and low temporal resolution, because of a limited acquisition time and possible through-plane geometrical distortion, since scanner software typically only corrects in-plane distortion. This last point has been addressed by some authors who have reported application of an offline 3D distortion correction to 2D MRI.^18^

Three-dimensional acquisition schemes do not have the limitations of 2D acquisition schemes and resulting 4D MRI could be of higher quality. Yet translating methods used in 2D acquisition schemes^12,13,16,18,20,25^ to 3D acquisition schemes is challenging because dynamic 3D MRI cannot typically be acquired with sufficient spatiotemporal resolution. However, good-quality 4D MRI has been obtained using advanced offline-reconstructions of highly undersampled data acquired using 3D non-Cartesian sequences.^15,26–29^

Alternatively, 4D MRI can be generated by applying a motion model to a reference volume.^30^ Blackall et al^31^ obtained 4D MRI by applying a single-parameter motion model, with motion vector fields (MVFs) extracted from intraoperative ultrasound images, to static MRI. In likewise methods, McClelland et al^32^ developed a temporal-fitting motion model for CT, which was based on performing deformable image registration between reference and free breathing volumes; Marx et al^33^ generated 4D CT by applying the motion information from 4D MRI to 3D CT. Recently, Stemkens et al^34^ proposed a motion model to generate real-time 4D MRI, where a set of MVFs were obtained based on dynamic 2D MRI and a reference volume.

To our knowledge, no method or motion model has been applied to generate 4D-T2w MRI from data measured with a 3D acquisition scheme. This might be because it is difficult to acquire dynamic 3D-T2w MRI, due to the long echo and repetition time required to achieve T2w contrast. Obtaining T2w MRI for lung RTP and guidance is important because T2w MRI is sensitive to both lung infiltrates and lesions with fluid content.^35^ Furthermore, T2w MRI enables an improved visualization of both mobile organs at risk (OARs) and tumor sites when compared with T1-weighted (T1w) MRI for cases such as esophageal cancer.^36^

In this study, we (1) introduce the MVF projection (MVFP) method, which provides a workflow to generate 4D-T2w MRI by applying the motion information from a 4D-T1w volume to a 3D-T2w volume; (2) verify calculated 4D-T2w MRI by comparing diaphragm positions, anatomical landmarks, and volumetric image similarity in generated 4D-T2w MRI to 4D-T1w MRI; and (3) discuss examples where 4D-T2w MRI more clearly shows tumor position, structure, and extent when compared with 4D-T1w MRI.

## MATERIALS AND METHODS

### Data Acquisition

Ten patients with non–small cell lung cancer (6 female, 4 male; aged, 63–86 years; 5 squamous cell carcinoma and 5 adenocarcinoma) were scanned with an axial 3D-T1w stack-of-stars spoiled gradient echo sequence in free breathing with golden angle spacing^37,38^ and an axial 3D-T2w turbo spin echo sequence^39^ with respiratory gating to exhalation at 1.5 T (MAGNETOM Aera; Siemens Healthcare, Erlangen, Germany). Axial orientation was chosen to facilitate delineation for the purpose of RTP. The T2w sequence was gated to exhalation using a liver dome navigator.^40^ The T1w sequence utilized a radial encoding scheme in the readout plane and a Cartesian slice encoding scheme. Each consecutive radial plane was obtained after rotating by the golden angle (θ ≈ 111.25 degrees).^38^

A range of sequence parameters were used due to variation in patient habitus. Initially, a relatively high bandwidth (1085 Hz) was selected (patients 1 to 6) but was later found to be suboptimal regarding image quality of reconstructed 4D-T1w MRI. After protocol optimization, a lower bandwidth (630 Hz) was used (patients 7 to 10), which resulted in an incremental improvement in image quality of 4D-T1w MRI and also enabled a smaller voxel size. Detailed acquisition parameters are listed in Table 1.

**TABLE 1:**
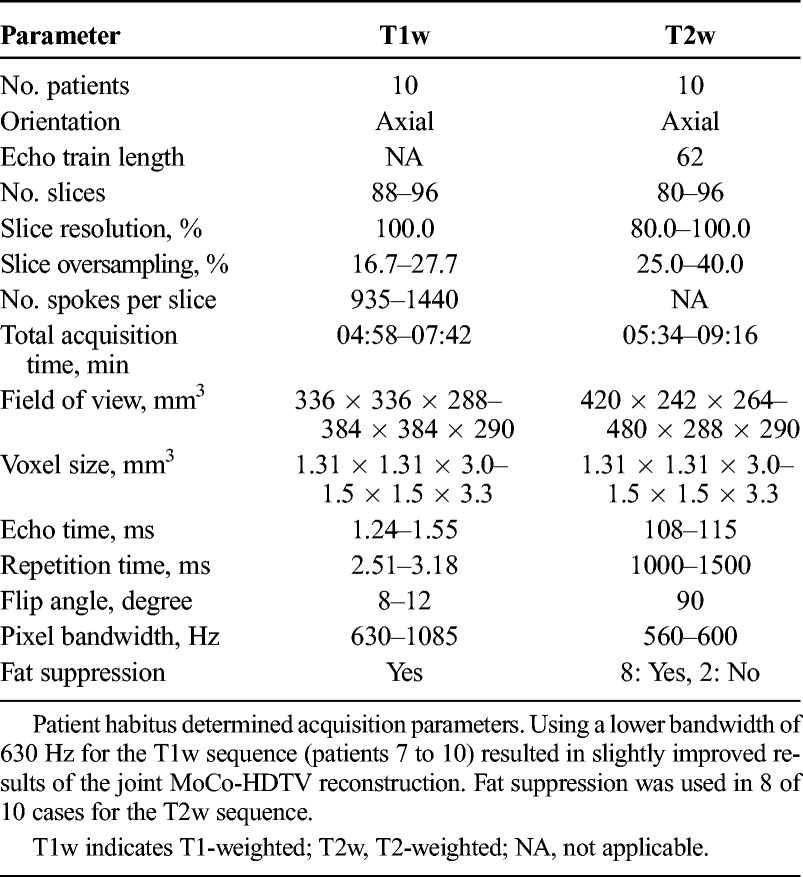
Acquisition Parameters of the T1w and T2w Sequences

### Reconstruction of T1w Data

The acquired data were retrospectively reconstructed using the 4D joint motion-compensated high-dimensional total variation (4D joint MoCo-HDTV) algorithm.^15^ Before reconstruction, an adaptive gradient-delay compensation was applied to the raw data, so that artifacts associated with inaccuracies in the timing of gradients were reduced.^41^ Afterward, the raw data were sorted into 20 overlapping respiratory phases based on the amplitude of the self-gating respiratory surrogate signal. The self-gating signal was extracted from the magnitude of the 9 central k-space points on each radial spoke that passed through the k-space center. Weightings of the HDTV operator were optimized by reducing temporal regularisation such that the images remained clear from undersampling artifacts while avoiding over-regularization of true motion. Subsequently, an offline gradient nonlinearity distortion correction was applied to each respiratory phase of the reconstructed 4D-T1w volume, using a spherical harmonics deconvolution method.^42,43^

### Overview of the MVF Projection Method

**FIGURE 1:**
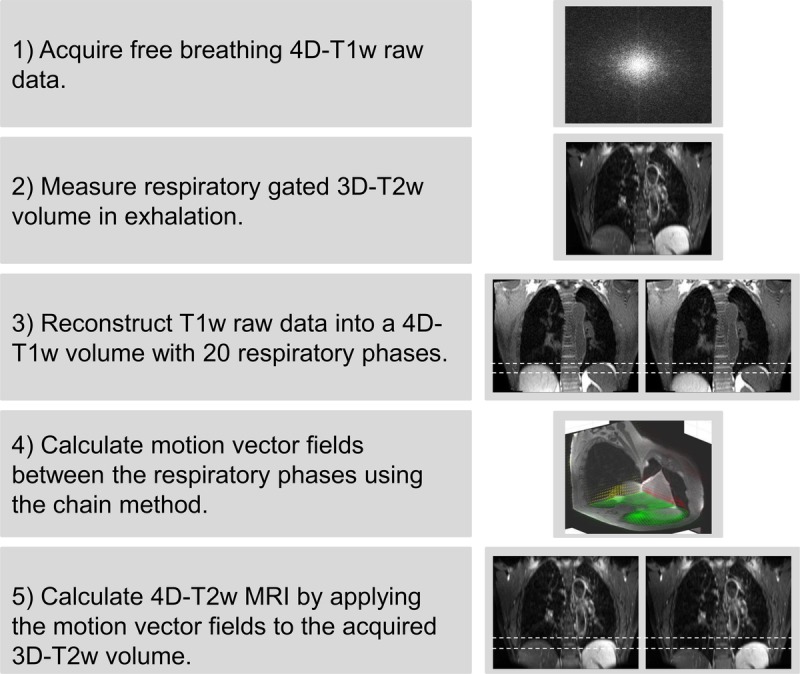
A visual overview of the motion vector field projection method. Note that the green, red, and yellow arrows in step 4 display the motion vector fields corresponding to the shown axial, coronal, and sagittal planes. Dashed white lines aid visualization of the diaphragm position on T1w and T2w MRI.

The MVFP method generates 4D-T2w MRI by extracting motion information from 4D-T1w MRI and applying it, using a chain method, to 3D-T2w MRI. An overview of the MVFP method is displayed in Figure 1.

### Calculation and Application of MVFs

A one-dimensional signal describing image similarity was obtained by calculating the normalized mutual information (NMI)^44^ (calculated in-plane and averaged over all slices) between the 3D-T2w volume and each respiratory phase j contained in the 4D-T1w volume; where *j* ∈ N (total number) respiratory phases. The respiratory phase *i* of the 3D-T2w volume was set equal to the respiratory phase *j* that corresponded to the signal maximum. In this way, a tie-phase was established between the 4D-T1w and 3D-T2w volumes.

Motion vector fields between the respiratory phases *i* and *j* of the reconstructed volumes (*T*1_*i*_, *T*1_*j*_) were calculated by deformable image registration. A b-spline GPU accelerated implementation of NiftyReg^45,46^ was used to calculate all deformable image registrations.

**FIGURE 2:**
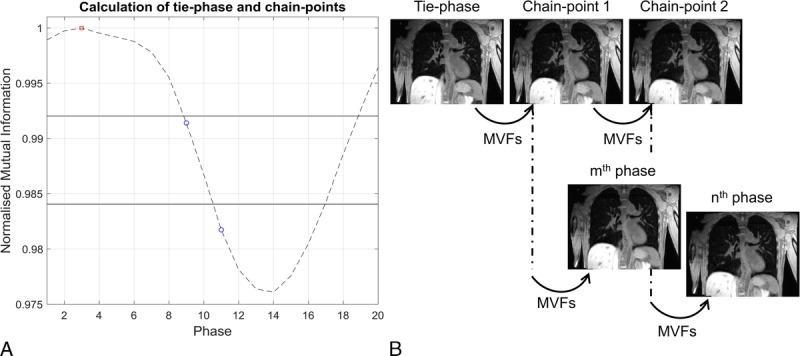
A, Displays the image similarity signal with the tie-point phase (red square) and chain-point phases (blue circles). The values (horizontal solid lines) of the fractions of the signal range, used to calculate the chain-point phases, are shown. B, The volumes corresponding to the chain-point phases are determined by sequentially warping the tie-phase with the required motion vector fields. The chain-point phases are used as a starting point to generate any arbitrary phase. For instance, the m^th^ phase is calculated with 2 deformations (tie-phase → chain-point 1; chain-point 1 → m^th^ phase) and the n^th^ phase requires 3 deformations (tie-phase → chain-point 1; chain-point 1 → chain-point 2; chain-point 2 → n^th^ phase).

A chain method, similar to that proposed by Boldea et al,^47^ was developed where the 3D-T1w volume at the n^th^ phase 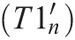 was obtained by sequentially applying a number of smaller deformations that are linked together at calculated chain-point phases, to the tie-phase (*T*1_*i*_). The chain method enables a balance between errors resulting from large deformations and concatenation of sequential deformations. An overview of the chain method can be found in Figure 2.

The chain-point phases were calculated based on fractions of the calculated image similarity signal range. This is possible because the image similarity signal indirectly corresponds to the deformation size between phases. The chain-point phases corresponding to inspiration were set equal to those of expiration, which is feasible because of the symmetry present in the respiratory pattern. A maximum of 3 deformations were chosen, and phases closest to 33% and 67% of the signal range were set as chain-points.

The parameters of NiftyReg were optimized by comparing the estimated (*T*1′) and reconstructed (*T*1) 4D-T1w volumes. The root mean square error (RMSE), dice coefficient (averaged over all respiratory phases), and visual image quality were analyzed as metrics. An NMI cost function with number of levels performed = 3, control point spacing = 3 mm, bending energy weighting = 0, Jacobian penalty weighting = 0, and maximum iterations = 500 was best able to reproduce the reconstructed 4D-T1w volume (dice > 0.965, RMSE < 5%, good qualitative agreement).

The 3D-T2w volume was registered and then interpolated to the matrix size of the 4D-T1w tie-phase. Calculated 4D-T2w MRI is then obtained by applying the calculated MVFs, using the chain method, to the registered and interpolated 3D-T2w volume.

All calculations were undertaken on an Intel Xeon E5-1660 processor with 8 cores at 3 GHz and 64 GB of memory.

### Verification of the 4D-T2w Volumes

The calculated 4D-T2w volumes were verified against their corresponding 4D-T1w volumes. Using MATLAB (The MathWorks, Natick, MA), a semiautomated edge-detection method was developed and used to verify the diaphragm positions, a radiation oncologist manually delineated control points for comparison of anatomical positions and the NMI was calculated to assess volumetric image similarity. For each metric, the differences between 4D-T1w and 4D-T2w MRI were compared with those between 4D-T1w and 3D-T2w MRI.

In the edge-detection method, the user manually places a rectangular 2D region of interest (ROI) over the right hemidiaphragm surface, orientated along the superior-inferior (SI) direction, on a coronal or sagittal slice. Along the SI dimension, the ROI should be sufficiently large to encompass expected respiratory motion (approximately 2−3 cm) and narrow in the medial-lateral dimension such that the bounded diaphragm surface is approximately flat. For consistency, an ROI was placed on the coronal slice that exhibited both the aortic arch and the descending aorta.

Intensity was measured along all SI lines within the 2D ROI. For each SI line, the diaphragm position was calculated by fitting a Gaussian to the derivative of the measured line intensity. To increase robustness, a ramp function was used. Outliers in diaphragm position across all SI lines were removed based on the interquartile range of the detected diaphragm positions. The diaphragm position was chosen as the mean of the remaining diaphragm positions within the 2D ROI and the standard deviation corresponds to the diaphragm width.

Using an in-house developed delineation toolkit, the radiation oncologist (6 years experience) identified 5 anatomical control points (1 static and 4 mobile) in even respiratory phases in both 4D-T1w and 4D-T2w MRI:

1. Posterior spinal canal at the superior aspect of T4.
2. Inferior point of the carina.
3. Bifurcation of the right middle and lower lobe bronchus.
4. Bifurcation of the left main bronchus and left upper lobe bronchus.
5. Right costophrenic angle.

**FIGURE 3:**
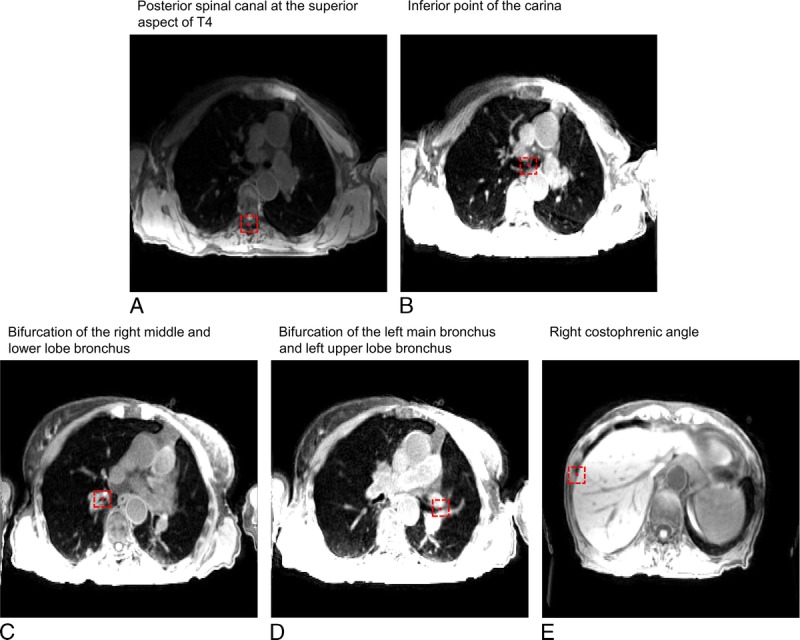
An example of control point delineation for patient 8 on a snapshot image of 4D-T1w MRI. The control points (red circles) are contained within dashed red boxes to assist visualization. The presented windowing scheme was optimized by the radiation oncologist for viewing of the anatomical landmark and not for surrounding anatomy.

The right costophrenic angle was delineated on coronal images, with the coronal slice chosen to correspond with the level of the bifurcation of the right upper and middle lobe bronchus. The delineation toolkit offered coronal, sagittal, and axial views and delineation could be performed on either of them. Figure 3 shows an example of control point delineation for patient 8. These particular control points were chosen because they are visible in both 4D-T1w and 4D-T2w MRI, as well as being reproducible across patient sets. To assess spatial coherence between landmarks, Euclidean distances were calculated between pairs of delineated points in both 4D-T1w and 4D-T2w MRI.

Normalized mutual information^44^ was calculated between each corresponding respiratory phase of the 4D-T1w and 4D-T2w volumes. The result was compared with the NMI measured between each respiratory phase of the 4D-T1w volume and the 3D-T2w volume.

If 4D-T1w and 4D-T2w MRI are commensurate in respiratory phase and geometrical position, then the NMI for all respiratory phases should be similar to the NMI assessed between the tie-phase of the 4D-T1w volume and the 3D-T2w volume.

## RESULTS

**FIGURE 4:**
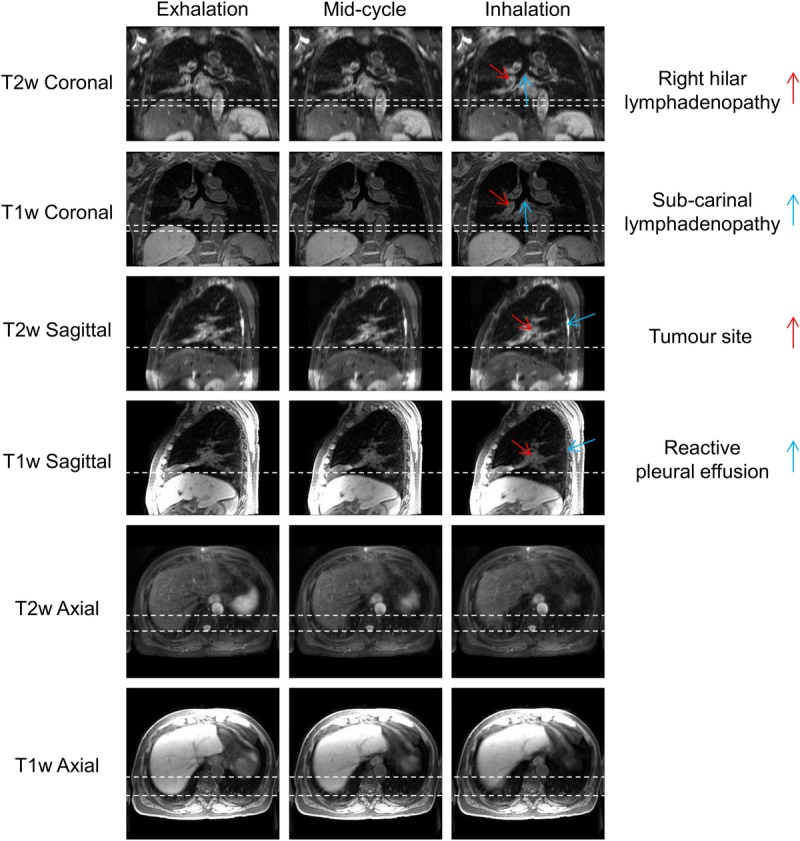
Example T1w and T2w coronal, sagittal, and axial views at exhalation, midcycle, and inhalation respiratory phases for patient 7. This patient was diagnosed with T1aN2 primary lung squamous cell carcinoma and had a small benign reactive pleural effusion. The dashed white lines aid visualization of the diaphragm surface position. The respiratory pattern of T1w MRI is preserved in T2w MRI. The tumor site, lymphadenopathy, and reactive pleural effusion are shown more clearly in T2w MRI than T1w MRI.

Four-dimensional T2w MRI was calculated for 10 patients with primary lung malignancies. Four-dimensional T1w MRI reconstruction, using a nonoptimized prototype implementation, took between 9 and 12 hours for 20 respiratory phases and the chain method took between 25 and 30 minutes. The mean period of the respiratory cycle averaged over patients was 4.1 ± 0.95 seconds. Figure 4 shows an example reconstructed 4D-T1w volume and a calculated 4D-T2w volume at respiratory phases corresponding to exhalation, midcycle, and inhalation. The movie in Supplemental Digital Content 1, http://links.lww.com/RLI/A316, displays a similar example, but with all respiratory phases. For all patients, 4D-T2w MRI exhibited qualitatively similar respiratory motion to corresponding 4D-T1w MRI.

### Verification of 4D-T2w MRI

**FIGURE 5:**
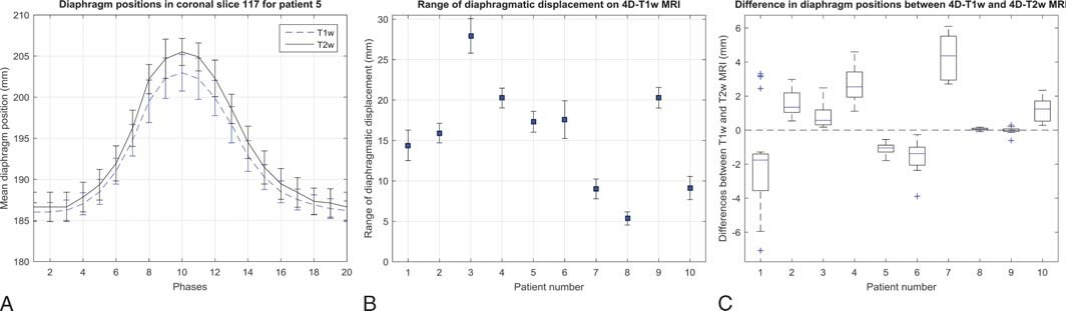
A, Example diaphragm surface positions, calculated using the edge-detection method, of the 4D-T1w and 4D-T2w volumes for patient 5. Error bars correspond to the standard deviation across SI lines and represent the width of the diaphragm surface. B, The ranges of diaphragmatic displacements in 4D-T1w MRI calculated using the edge-detection method. Errors bars show the uncertainty in the range. C, Box plot of the spread of the difference in diaphragm surface positions, between the 4D-T1w and 4D-T2w volumes, as calculated by the edge-detection method, for the 10 patients.

Mean diaphragm surface positions were calculated using the edge-detection method for all patients on both T1w and T2w images. Figure 5A displays an example of the diaphragm surface displacement for patient 5. Figure 5B shows the range of diaphragmatic displacement of 4D-T1w MRI, as calculated by the edge-detection method. Figure 5C shows the spread of the displacement between diaphragm positions on both T1w and T2w images across all respiratory phases. Median diaphragm positions were consistent with less than 1 slice thickness (3.3 mm) for all patients, except patient 7, which exhibited median displacements within 6.6 mm. For all patients, T1w and T2w MRI were less consistent at inhalation than at exhalation. The Pearson correlation coefficient was calculated between the median diaphragm differences of 4D-T1w and 4D-T2w MRI, and the range of diaphragmatic displacements in 4D-T1w MRI. No significant correlation (*r* = −0.19, *P* = 0.60) was observed.

There was a reduction in the mean and standard deviation, averaged over all patients, of the interquartile range of the differences in diaphragm positions over all respiratory phases, between 4D-T1w and 4D-T2w MRI (1.11 ± 0.81 mm) compared to 4D-T1w and 3D-T2w MRI (9.83 ± 3.95 mm).

**FIGURE 6:**
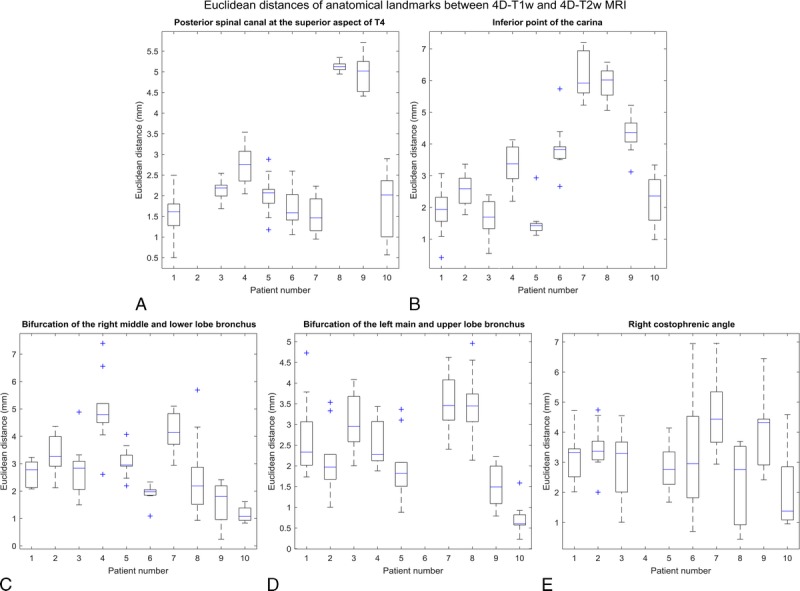
Box plot of spread of Euclidean distances, for all even respiratory phases, between pairs of anatomical landmarks that were delineated in both 4D-T1w and 4D-T2w MRI. All Euclidean distances agreed to less than 7.6 mm (Euclidean distance of 2 voxels) and 355 of 470 pairs of anatomical landmarks were consistent with less than 3.8 mm (Euclidean distance of 1 voxel).

A radiation oncologist manually delineated 5 anatomical landmarks on both 4D-T1w and 4D-T2w MRI, and the Euclidean distances between them were calculated. Results are shown in Figure 6.

Euclidean distances between all corresponding anatomical landmarks were within or better than 7.6 mm (Euclidean distance of 2 voxels) and less than 3.8 mm (Euclidean distance of 1 voxel) for 355 of 470 delineated pairs of anatomical control points. Three anatomical landmarks were excluded from delineation: for patient 2, the posterior spinal canal at the superior aspect of T4 was removed because of poor visibility on T2w MRI; for patient 6, the bifurcation of the left main bronchus and left upper lobe bronchus was omitted due to partial collapse of the left upper lobe; for patient 4, the right costophrenic angle was outside the acquired field of view.

There was a reduction in the mean and standard deviation, averaged over patients, of the interquartile range, calculated over respiratory phases, of the Euclidean distances for the mobile ROIs (ROIs, 2–5) between 4D-T1w and 4D-T2w MRI (0.80 ± 0.35 mm, 0.85 ± 0.39 mm, 0.78 ± 0.28 mm, 1.62 ± 0.70 mm) and between 4D-T1w and 3D-T2w MRI (1.23 ± 0.48 mm, 2.02 ± 0.98 mm, 1.19 ± 0.94 mm, 5.16 ± 2.08 mm).

**FIGURE 7:**
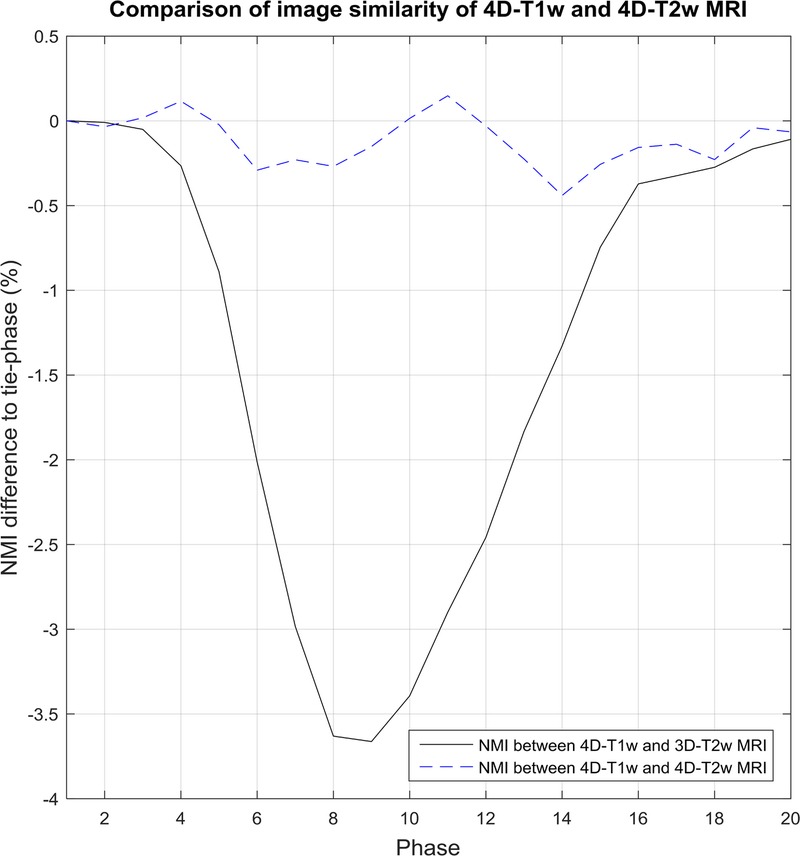
Shows an example comparison for patient 10, where results are relative to the normalized mutual information (NMI) calculated between the tie-phase of 4D-T1w and 3D-T2w MRI. In this case, the tie-phase is respiratory phase 1. NMI between 4D-T1w and 3D-T2w MRI (black solid line) indirectly corresponds to a respiratory signal. This pattern is not observed when examining NMI between 4D-T1w and 4D-T2w MRI (blue dashed curve).

The NMI was calculated between corresponding respiratory phases of 4D-T1w and 4D-T2w MRI, and was compared with the NMI of 4D-T1w and 3D-T2w MRI. Comparisons were made in relation to the NMI calculated between the tie-phase of 4D-T1w and 3D-T2w MRI. Figure 7 shows an example comparison for patient 10. Percentage differences in NMI (mean and standard deviation, calculated over all respiratory phases and patients) of 4D-T1w and 4D-T2w MRI were 0.41% ± 0.37% and between 4D-T1w and 3D-T2w MRI were −1.82% ± 1.76%.

### Image Artifacts

**FIGURE 8:**
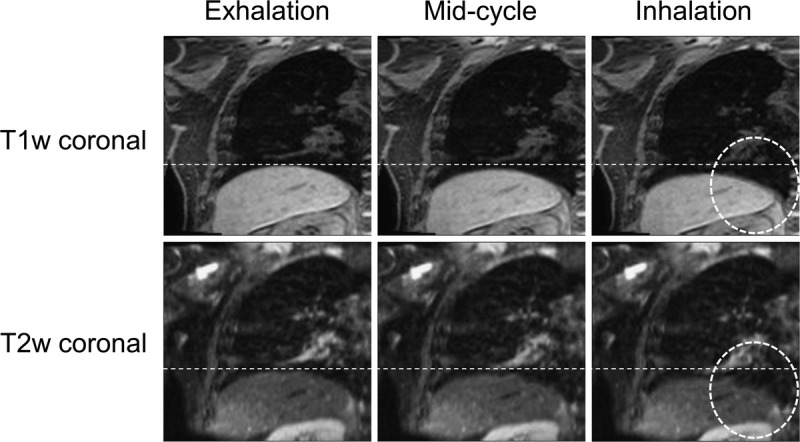
An example of the deformation artifact for patient 7. In the T2w snapshots, a deformation of the right hemidiaphragm boundary is seen at midcycle and a comparatively large deformation at inhalation (dashed circle). The deformation is worse at inhalation than at midcycle and is not displayed in the corresponding T1w snapshots.

The dominant image artifact observed after application of the MVFP method was associated with inaccuracies in deformable image registration, which led to a reduction in the quality of 4D-T2w MRI for all patients and was found to increase in magnitude with deformation size. An example is shown in Figure 8.

Artifacts that were already present in 3D-T2w MRI were propagated into 4D-T2w MRI. For all patients, minor ghosting was displayed and for 2 patients intensity inhomogeneity was apparent.

### Clinical Evaluation

The radiation oncologist reported improved tumor definition in 4D-T2w MRI when compared with 4D-T1w MRI. Furthermore, important clinical information is displayed. In the case presented in Figure 4, T2w MRI better highlights extent and position of reactive pleural effusion and the lymphadenopathy than T1w MRI.

**FIGURE 9:**
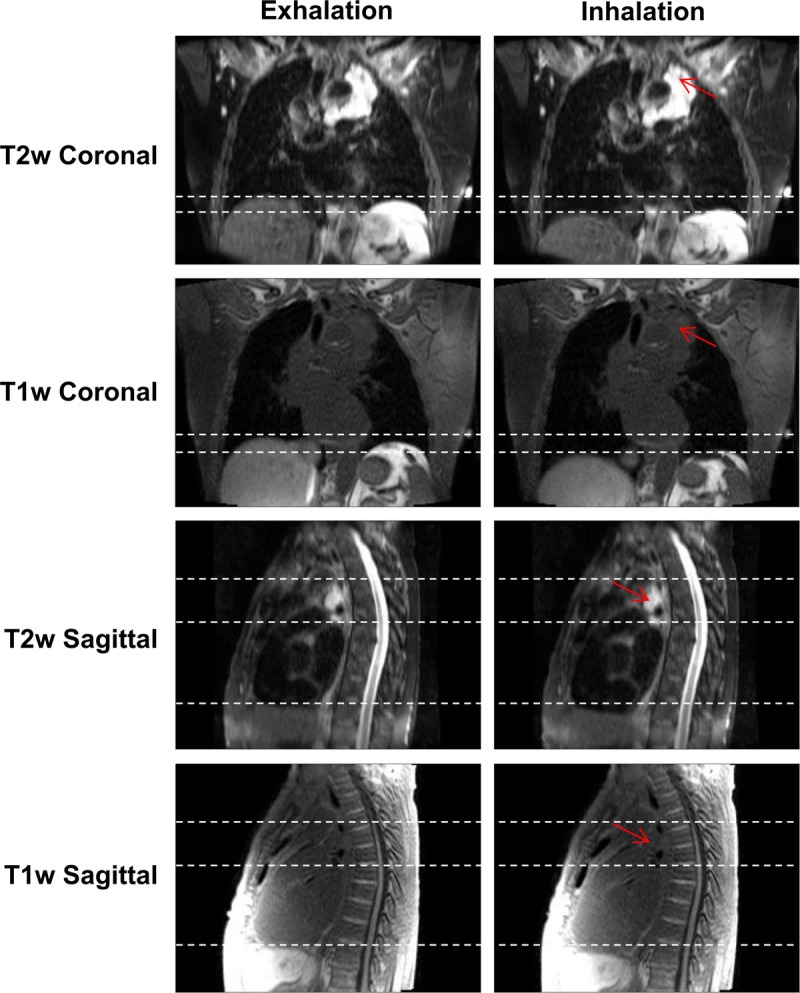
Snapshots of 4D-T1w and 4D-T2w MRI at the exhalation and inhalation respiratory phases for patient 3, who was diagnosed with T4N2 adenocarcinoma. The tumor is radically treatable, but is embedded around the heart and oesophagus. Unlike T1w MRI, T2w MRI displays a high tumor-tissue contrast enabling tumor position and structure to be clearly delineated. Sliding motion of the tumor site against the chest wall is displayed in the sagittal plane and is more clearly presented in T2w than T1w MRI. Red arrows point to the tumor site and dashed white lines aid assessment of both superior-inferior diaphragm and sliding motion.

In some situations, 4D-T2w MRI was particularly advantageous when compared with 4D-T1w MRI, for instance when mobile tumor beds were attached to or adjacent to OARs, as demonstrated in Figure 9 and in the movie provided as Supplemental Digital Content 2, http://links.lww.com/RLI/A317. In the T1w images, the tumor-tissue contrast is low and it is challenging to delineate the tumor. However, the tumor extent and structure is clearly visible in T2w MRI. Furthermore, the anterior part of the tumor site is sliding nonrigidly against the chest-wall and the extent of attachment and sliding motion is more easily visualized in T2w MRI than T1w MRI.

## DISCUSSION

### Pulse Sequences

A sequence with a radial trajectory and golden angle spacing was selected to acquire the T1w data because of advantages in image quality, such as incoherent aliasing and insensitivity to motion^37^ and because a self-gating signal could be obtained through frequent measurements of the k-space center.^48^ Alternative non-Cartesian trajectories could be used,^26^ but a stack-of-stars approach is more efficient during reconstruction, as a Fourier transform can be applied along the slice direction.^37^

We used an incremental improvement during the study in reducing the readout bandwidth of the 4D-T1w MRI acquisition. However, there was no significant difference in median diaphragm displacements on T1w and T2w MRI between patients 1 to 6 and patients 7 to 10 (unpaired 2-tailed *t* test, significance level α = 0.05, *P* = 0.34). Therefore, a group analysis was justified.

The chosen T2w sequence uses a variable flip angle distribution to extend the echo train and speed up acquisition, enabling volumetric T2w MRI to be acquired in a clinically acceptable period (5–9 minutes). The application of a 3D acquisition is preferable to 2D slice-selective excitation sequences, commonly used in alternative 4D MRI methods,^12,13,16,18,20,25^ because the resulting MRI provides improved SNR. This property enables thinner slices and consequently a reduction in stair-case artifacts due to highly nonisotropic voxel sizes. Yet, in Du et al,^13^ 4D-T2w MRI was calculated with a similar slice thickness of 3 mm, but a comparatively smaller field of view along the slice-direction was acquired. Furthermore, using 3D sequences might improve image quality, for delineation purposes, by reducing the influence of a complex magnetization history (impact of in-flow, slice-selection pulses, excitation frequency). Primarily, 3D sequences offer improved geometrical fidelity when using 3D distortion correction, which on commercially available systems is often not available for 2D sequences, but nonetheless is essential for RTP.

### Image Reconstruction

The 4D joint MoCo-HDTV reconstruction was chosen because it not only enables high undersampling factors by using 100% of the raw data for reconstruction of each respiratory phase but also results in good image quality with comparatively low streaking artifacts and high sharpness.^15^ There are alternative reconstructions that utilize similar methods but are not yet readily available for the whole thorax.^49^

### Artifacts Apparent Using the MVF Projection Method

The MVFP method has limitations. The deformation artifact causes the diaphragm surface to be discontinuous and predominantly occurs at inhalation. A similar artifact has been reported when using NiftyReg with lung 4D CT data.^50^ In our case, the artifact is present in the estimated 4D-T1w volume (*T*1′) but not in the reconstructed 4D-T1w volume (*T*1) and, therefore, is due to errors accumulated during application of the chain method. The extent of the artifact can be loosely quantified as the interquartile range of the difference in diaphragm positions between 4D-T1w and 4D-T2w MRI; the mean (calculated over all patients) of which is 1.11 mm (less than 0.5 voxel), demonstrating that the artifact has only a minor impact on diaphragm continuity of 4D-T2w MRI.

### Verification

Four-dimensional T2w MRI was verified with respect to 4D-T1w MRI. Another option might have been to compare the inhalation respiratory phase of 4D-T2w MRI to T2w MRI acquired in breath-hold. However, this approach was not pursued because inhalation in breath-hold can be deeper than in free-breathing.^30,51^

### Diaphragm Positions

The median diaphragm positions were consistent with less than 6.6 mm (2 voxels) for all 10 patients and less than 3.3 mm (1 voxel) for 9 of 10 patients. Furthermore, the mean of the interquartile range of observed differences between 4D-T1w and 4D-T2w MRI was ≈ 8.7 mm smaller than between 4D-T1w and 3D-T2w MRI, demonstrating that calculated 4D-T2w MRI not only contained similar motion information to 4D-T1w MRI but was also spatially coherent.

The observed differences are partly due to a mismatch in respiratory phase between 4D-T1w and 3D-T2w MRI; as for all patients, comparison of the diaphragm positions (using the edge-detection method) in 4D-T1w and 3D-T2w MRI indicated that no exact match could be found. This might be solved by nonrigidly warping the 3D-T2w volume to the closest matching phase of the 4D-T1w volume. One hypothesis was that the differences depended on the magnitude of the diaphragm displacement during the respiratory cycle, but no significant correlation was found.

The larger differences observed for patient 7 might result from a collapse of the middle lobe of the right lung, as can be seen in the movie of Supplemental Digital Content 1, http://links.lww.com/RLI/A316.

### Anatomical Control Points

Four-dimensional T2w MRI was anatomically similar to 4D-T1w MRI because all Euclidean distances, calculated between corresponding control points, were consistent with less than 7.6 mm (Euclidean distance of 2 voxels) and Euclidean distances of 355 of 470 pairs of control points agreed to less than 3.8 mm (Euclidean distance of one voxel). Applying the MVFP method led to improved similarity of T1w and T2w MRI as the mean interquartile range of Euclidean distances between mobile ROIs of 4D-T1w and 4D-T2w MRI were smaller than those between 4D-T1w and 3D-T2w MRI by ≈ 1.39 mm.

Verification by manual delineation is limited, because it is a subjective process such that part of the presented differences, between pairs of anatomical control points, could be attributed to user dependence. Multiple observers might increase the accuracy of determined anatomical landmark positions.

A single observer approach was undertaken to reflect likely clinical practice, which could involve manual delineation on several respiratory phases.

### Volumetric Image Similarity

A high volumetric image similarity was observed between 4D-T1w and 4D-T2w MRI, as NMI, relative to the tie-phase of 4D-T1w and 3D-T2w MRI, was coherent within 0.41% ± 0.37%. No dependence on respiration of the NMI calculated between corresponding respiratory phases of 4D-T1w and 4D-T2w MRI is apparent in Figure 7. Four-dimensional T2w MRI is thus commensurate to 4D-T1w MRI in spatiotemporal location.

A drawback of this approach is that the NMI metric is not only sensitive to relevant spatiotemporal information but also to image artifacts and noise. This metric was chosen because it can handle analysis between different image contrasts.^44^

### Application

As a proof-of-principle, high-quality geometrically accurate 4D-T2w images were calculated and could assist clinicians in obtaining RTPs for anatomical regions affected by respiratory motion by improving tumor definition compared with 4D-T1w or 4D CT images. For instance, calculated MVFs might be applied to the 3D-T2w volume to generate midvent T2w MRI, which could be used alongside midvent CT,^2^ to aid delineation of malignant tissue. The current workflow for radiotherapy planning and delineation includes a free breathing ^18^F-fluorodexoyglucose position emission tomography scan. Four-dimensional position emission tomography imaging has been proposed, but further clinical validation is required,^52^ and unlike the presented 4D-T2w MRI, it has limited spatial resolution (5–7 mm).^53,54^

The improved tumor-tissue contrast exhibited by 4D-T2w MRI could be particularly beneficial when moving tumor sites are adjacent to OARs, because it can often be challenging to delineate such sites on 4D-T1w MRI or 4D CT.^36,55^ In addition, 4D-T2w MRI could improve the reliability and specificity of assessment of chest wall invasion when compared with 4D-T1w MRI and 4D CT.^56^

Four-dimensional T2w MRI could be used as part of an MR-only workflow for hybrid MR-IGRT systems.^4–8^ For instance, high-quality 4D-T2w MRI could act as a reference volume for both retrospective evaluation of the delivered treatment and the generation of real-time 4D-T2w MRI for beam-on guidance and planning, in methods as proposed by Stemkens et al.^34^

The MVFP method is not limited to 4D-T2w MRI and could be applied to simulate 4D MRI displaying any required contrast. For specific contrasts, such as diffusion-weighted MRI or ultrashort echo time imaging, it is not possible to use a stack-of-stars k-space sampling,^57^ making it more difficult to apply state-of-the-art 4D reconstruction methods. In this case, the MVFP method could act as a solution to generate high-quality 4D MRI.

## CONCLUSIONS

Four-dimensional T2w MRI was calculated retrospectively by applying the motion information from a 4D-T1w volume to a static 3D-T2w volume. Good quality geometrically accurate 4D-T2w volumes were obtained, providing high temporal resolution. Four-dimensional T2w MRI may assist clinicians in delineating lesions within volumes affected by respiratory motion that are challenging to outline on a 4D-T1w volume, making it a promising candidate for applications in radiotherapy, particularly with hybrid MR-IGRT systems in mind.

## Supplementary Material

SUPPLEMENTARY MATERIAL

## References

[1] Keall PJ Mageras GS Balter JM The management of respiratory motion in radiation oncology report of AAPM Task Group 76. *Med Phys*. 2006;33:3874–3900. 17089851 10.1118/1.2349696

[2] Wolthaus JW Sonke JJ van Herk M Comparison of different strategies to use four-dimensional computed tomography in treatment planning for lung cancer patients. *Int J Radiat Oncol Biol Phys*. 2008;70:1229–1238. 18313530 10.1016/j.ijrobp.2007.11.042

[3] Sonke JJ Zijp L Remeijer P Respiratory correlated cone beam CT. *Med Phys*. 2005;32:1176–1186. 15895601 10.1118/1.1869074

[4] Raaymakers BW Lagendijk JJ Overweg J Integrating a 1.5 T MRI scanner with a 6 MV accelerator: proof of concept. *Phys Med Biol*. 2009;54:N229–N237. 19451689 10.1088/0031-9155/54/12/N01

[5] Lagendijk JJ Raaymakers BW Van den Berg CA MR guidance in radiotherapy. *Phys Med Biol*. 2014;59:R349–R369. 25322150 10.1088/0031-9155/59/21/R349

[6] Fallone BG The rotating biplanar linac-magnetic resonance imaging system. *Semin Radiat Oncol*. 2014;24:200–202. 24931093 10.1016/j.semradonc.2014.02.011

[7] Mutic S Dempsey JF The ViewRay system: magnetic resonance-guided and controlled radiotherapy. *Semin Radiat Oncol*. 2014;24:196–199. 24931092 10.1016/j.semradonc.2014.02.008

[8] Thwaites D Keall P Holloway L Observations on MR-LINAC systems and rationale for MR-Linac use: the Australian MR-Linac project as an example. *Phys Medica*. 2014;30:e25.

[9] Schmidt MA Payne GS Radiotherapy planning using MRI. *Phys Med Biol*. 2015;60:R323–R361. 26509844 10.1088/0031-9155/60/22/R323 PMC5137785

[10] Weygand J Fuller CD Ibbott GS Spatial precision in magnetic resonance imaging-guided radiation therapy: the role of geometric distortion. *Int J Radiat Oncol Biol Phys*. 2016;95:1304–1316. 27354136 10.1016/j.ijrobp.2016.02.059

[11] Hofmann M Pichler B Schölkopf B Towards quantitative PET/MRI: a review of MR-based attenuation correction techniques. *Eur J Nucl Med Mol Imaging*. 2009;36(suppl 1):S93–S104. 19104810 10.1007/s00259-008-1007-7

[12] Hu Y Caruthers SD Low DA Respiratory amplitude guided 4-dimensional magnetic resonance imaging. *Int J Radiat Oncol Biol Phys*. 2013;86:198–204. 23414769 10.1016/j.ijrobp.2012.12.014 PMC3628273

[13] Du D Caruthers SD Glide-Hurst C High-quality T2-weighted 4-dimensional magnetic resonance imaging for radiation therapy applications. *Int J Radiat Oncol Biol Phys*. 2015;92:430–437. 25838186 10.1016/j.ijrobp.2015.01.035 PMC4431950

[14] Larson AC Kellman P Arai A Preliminary investigation of respiratory self-gating for free-breathing segmented cine MRI. *Magn Reson Med*. 2005;53:159–168. 15690515 10.1002/mrm.20331 PMC1939886

[15] Rank CM Heußer T Buzan MT 4D respiratory motion-compensated image reconstruction of free-breathing radial MR data with very high undersampling. *Magn Reson Med*. 2017;77:1170–1183. 26991911 10.1002/mrm.26206

[16] Cai J Chang Z Wang Z Four-dimensional magnetic resonance imaging (4D-MRI) using image-based respiratory surrogate: a feasibility study. *Med Phys*. 2011;38:6384–6394. 22149822 10.1118/1.3658737 PMC4108683

[17] King AP Buerger C Tsoumpas C Thoracic respiratory motion estimation from MRI using a statistical model and a 2-D image navigator. *Med Image Anal*. 2012;16:252–264. 21959365 10.1016/j.media.2011.08.003

[18] Tryggestad E Flammang A Han-Oh S Respiration-based sorting of dynamic MRI to derive representative 4D-MRI for radiotherapy planning. *Med Phys*. 2013;40:051909. 23635279 10.1118/1.4800808

[19] Savill F Schaeffter T King AP Assessment of input signal positioning for cardiac respiratory motion models during different breathing patterns. *IEEE International Symposium on Biomedical Imaging: From Nano to Macro*. 2011:1698–1701.

[20] Liu Y Yin FF Czito BG T2-weighted four dimensional magnetic resonance imaging with result-driven phase sorting. *Med Phys*. 2015;42:4460–4471. 26233176 10.1118/1.4923168 PMC4491020

[21] Vedam SS Keall PJ Kini VR Acquiring a four-dimensional computed tomography dataset using an external respiratory signal. *Phys Med Biol*. 2002;48:45–62. 10.1088/0031-9155/48/1/304 12564500

[22] Hui C Wen Z Stemkens B 4D MR imaging using robust internal respiratory signal. *Phys Med Biol*. 2016;61:3472–3487. 27049817 10.1088/0031-9155/61/9/3472

[23] Li G Wei J Olek D Direct comparison of respiration-correlated four-dimensional magnetic resonance imaging reconstructed using concurrent internal navigator and external bellows. *Int J Radiat Oncol Biol Phys*. 2017;97:596–605. 28011048 10.1016/j.ijrobp.2016.11.004 PMC5288126

[24] Koch N Liu HH Starkschall G Evaluation of internal lung motion for respiratory-gated radiotherapy using MRI: part I–correlating internal lung motion with skin fiducial motion. *Int J Radiat Oncol Biol Phys*. 2004;60:1459–1472. 15590177 10.1016/j.ijrobp.2004.05.055

[25] Liu Y Yin FF Chen NK Four dimensional magnetic resonance imaging with retrospective k-space reordering: a feasibility study. *Med Phys*. 2015;42:534–541. 25652474 10.1118/1.4905044 PMC4288543

[26] Deng Z Pang J Yang W Four-dimensional MRI using three-dimensional radial sampling with respiratory self-gating to characterize temporal phase-resolved respiratory motion in the abdomen. *Magn Reson Med*. 2016;75:1574–1585. 25981762 10.1002/mrm.25753 PMC4644523

[27] Feng L Axel L Chandarana H XD-GRASP: golden-angle radial MRI with reconstruction of extra motion-state dimensions using compressed sensing. *Magn Reson Med*. 2016;75:775–788. 25809847 10.1002/mrm.25665 PMC4583338

[28] Feng L Grimm R Block KT Golden-angle radial sparse parallel MRI: combination of compressed sensing, parallel imaging, and golden-angle radial sampling for fast and flexible dynamic volumetric MRI. *Magn Reson Med*. 2014;72:707–717. 24142845 10.1002/mrm.24980 PMC3991777

[29] Mickevicius NJ Paulson ES Investigation of undersampling and reconstruction algorithm dependence on respiratory correlated 4D-MRI for online MR-guided radiation therapy. *Phys Med Biol*. 2016;62:2910–2921. 27997382 10.1088/1361-6560/aa54f2

[30] McClelland JR Hawkes DJ Schaeffter T Respiratory motion models: a review. *Med Image Anal*. 2013;17:19–42. 23123330 10.1016/j.media.2012.09.005

[31] Blackall JM Penney GP King AP Alignment of sparse freehand 3-D ultrasound with preoperative images of the liver using models of respiratory motion and deformation. *IEEE Trans Med Imaging*. 2005;24:1405–1416. 16279078 10.1109/TMI.2005.856751

[32] McClelland JR Blackall JM Tarte S A continuous 4D motion model from multiple respiratory cycles for use in lung radiotherapy. *Med Phys*. 2006;33:3348–3358. 17022231 10.1118/1.2222079

[33] Marx M Ehrhardt J Werner R Simulation of spatiotemporal CT data sets using a 4D MRI-based lung motion model. *Int J Comput Assist Radiol Surg*. 2014;9:401–409. 24323401 10.1007/s11548-013-0963-y

[34] Stemkens B Tijssen RH de Senneville BD Image-driven, model-based 3D abdominal motion estimation for MR-guided radiotherapy. *Phys Med Biol*. 2016;61:5335–5355. 27362636 10.1088/0031-9155/61/14/5335

[35] Biederer J Beer M Hirsch W MRI of the lung (2/3). Why … when … how? *Insights Imaging*. 2012;3:355–371. 22695944 10.1007/s13244-011-0146-8 PMC3481084

[36] Riddell AM Hillier J Brown G Potential of surface-coil MRI for staging of esophageal cancer. *AJR Am J Roentgenol*. 2006;187:1280–1287. 17056917 10.2214/AJR.05.0559

[37] Block KT Chandarana H Milla S Towards routine clinical use of radial stack-of-stars 3d gradient-echo sequences for reducing motion sensitivity. *J Korean Phys Soc*. 2014;18:87–106.

[38] Winkelmann S Schaeffter T Koehler T An optimal radial profile order based on the Golden Ratio for time-resolved MRI. *IEEE Trans Med Imaging*. 2007;26:68–76. 17243585 10.1109/TMI.2006.885337

[39] Lichy MP Wietek BM Mugler JP 3rd Magnetic resonance imaging of the body trunk using a single-slab, 3-dimensional, T2-weighted turbo-spin-echo sequence with high sampling efficiency (SPACE) for high spatial resolution imaging: initial clinical experiences. *Invest Radiol*. 2005;40:754–760. 16304477 10.1097/01.rli.0000185880.92346.9e

[40] Danias PG McConnell MV Khasgiwala VC Prospective navigator correction of image position for coronary MR angiography. *Radiology*. 1997;203:733–736. 9169696 10.1148/radiology.203.3.9169696

[41] Block K Uecker M Simple method for adaptive gradient-delay compensation in radial MRI. *ISMRM 19th Annual Meeting & Exhibition*. 2011;2816.

[42] Doran SJ Charles-Edwards L Reinsberg SA A complete distortion correction for MR images: I. Gradient warp correction. *Phys Med Biol*. 2005;50:1343–1361. 15798328 10.1088/0031-9155/50/7/001

[43] Janke A Zhao H Cowin GJ Use of spherical harmonic deconvolution methods to compensate for nonlinear gradient effects on MRI images. *Magn Reson Med*. 2004;52:115–122. 15236374 10.1002/mrm.20122

[44] Pluim JP Maintz JB Viergever MA Mutual-information-based registration of medical images: a survey. *IEEE Trans Med Imaging*. 2003;22:986–1004. 12906253 10.1109/TMI.2003.815867

[45] Modat M Ridgway GR Taylor ZA Fast free-form deformation using graphics processing units. *Comput Methods Programs Biomed*. 2010;98:278–284. 19818524 10.1016/j.cmpb.2009.09.002

[46] Rueckert D Sonoda LI Hayes C Nonrigid registration using free-form deformations: application to breast MR images. *IEEE Trans Med Imaging*. 1999;18:712–721. 10534053 10.1109/42.796284

[47] Boldea V Sharp GC Jiang SB 4D-CT lung motion estimation with deformable registration: quantification of motion nonlinearity and hysteresis. *Med Phys*. 2008;35:1008–1018. 18404936 10.1118/1.2839103

[48] Buehrer M Curcic J Boesiger P Prospective self-gating for simultaneous compensation of cardiac and respiratory motion. *Magn Reson Med*. 2008;60:683–690. 18727084 10.1002/mrm.21697

[49] Aitken AP Henningsson M Botnar RM 100% Efficient three-dimensional coronary MR angiography with two-dimensional beat-to-beat translational and bin-to-bin affine motion correction. *Magn Reson Med*. 2015;74:756–764. 25236813 10.1002/mrm.25460

[50] Modat M McClelland J Ourselin S Lung registration using the NiftyReg package. *Medical Image Analysis for the Clinic-A Grand Challenge*. 2010;2010:33–42.

[51] Blackall JM Ahmad S Miquel ME MRI-based measurements of respiratory motion variability and assessment of imaging strategies for radiotherapy planning. *Phys Med Biol*. 2006;51:4147–4169. 16912374 10.1088/0031-9155/51/17/003

[52] Konert T Vogel W MacManus MP PET/CT imaging for target volume delineation in curative intent radiotherapy of non-small cell lung cancer: IAEA consensus report 2014. *Radiother Oncol*. 2015;116:27–34. 25869338 10.1016/j.radonc.2015.03.014

[53] Kumar S Liney G Rai R Magnetic resonance imaging in lung: a review of its potential for radiotherapy. *Br J Radiol*. 2016;89:20150431. 26838950 10.1259/bjr.20150431 PMC4846194

[54] Hanna GG van Sörnsen de Koste JR Dahele MR Defining target volumes for stereotactic ablative radiotherapy of early-stage lung tumours: a comparison of three-dimensional 18 F-fluorodeoxyglucose positron emission tomography and four-dimensional computed tomography. *Clin Oncol (R Coll Radiol)*. 2012;24:e71–e80. 22445302 10.1016/j.clon.2012.03.002

[55] Brown G Daniels IR Richardson C Techniques and trouble-shooting in high spatial resolution thin slice MRI for rectal cancer. *Br J Radiol*. 2005;78:245–251. 15730990 10.1259/bjr/33540239

[56] Khalil A Majlath M Gounant V Contribution of magnetic resonance imaging in lung cancer imaging. *Diagn Interv Imaging*. 2016;97:991–1002. 27693089 10.1016/j.diii.2016.08.015

[57] Triphan SM Jobst BJ Breuer FA Echo time dependence of observed T1 in the human lung. *J Magn Reson Imaging*. 2015;42:610–616. 25604043 10.1002/jmri.24840

