# Supplementary material for: T2-Weighted 4D Magnetic Resonance Imaging for Application in Magnetic Resonance–Guided Radiotherapy Treatment Planning
Source: Invest Radiol. 2017 Apr 28;52(10):563–73. doi: 10.1097/RLI.0000000000000381 (PMC5581953; doi:10.1097/RLI.0000000000000381)
Supplement: SUPPLEMENTARY MATERIAL [file rli-52-563-s001.docx]

*T2 weighted 4D MRI for application in MR-guided radiotherapy treatment planning*

*Abstract*

*Objectives*

To develop and verify a method to obtain good temporal resolution T2 weighted 4D (4D-T2w) magnetic resonance imaging (MRI) by employing motion information from T1 weighted 4D (4D-T1w) MRI, to support treatment planning in MR-guided radiotherapy.

*Materials and Methods*

Ten patients with primary non-small cell lung cancer were scanned at 1.5 T axially with a volumetric T2 weighted turbo spin echo sequence gated to exhalation and a volumetric T1 weighted stack-of-stars spoiled gradient echo sequence with golden angle spacing acquired in free breathing. From the latter, 20 respiratory phases were reconstructed using the recently developed 4D joint MoCo-HDTV algorithm based on the self-gating signal obtained from the k-space centre. Motion vector fields describing the respiratory cycle were obtained by deformable image registration between the respiratory phases and projected onto the T2 weighted image volume. The resulting 4D-T2w volumes were verified against the 4D-T1w volumes: an edge-detection method was employed to measure the diaphragm positions, the locations of anatomical landmarks delineated by a radiation oncologist were compared and normalised mutual information (NMI) was calculated to evaluate volumetric image similarity.

*Results*

High-resolution 4D-T2w MRI was obtained. Respiratory motion was preserved on calculated 4D-T2w MRI, with median diaphragm positions being consistent to less than 6.6 mm (2 voxels) for all patients and less than 3.3 mm (one voxel) for nine out of ten patients. Geometrical positions were coherent between 4D-T1w and 4D-T2w MRI as Euclidean distances between all corresponding anatomical landmarks agreed to within 7.6 mm (Euclidean distance of two voxels) and are below 3.8 mm (Euclidean distance of one voxel) for 355 out of 470 pairs of anatomical landmarks. Volumetric image similarity was commensurate between 4D-T1w and 4D-T2w MRI, as mean percentage differences in NMI (calculated over all respiratory phases and patients), between corresponding respiratory phases of 4D-T1w and 4D-T2w MRI and the tie-phase of 4D-T1w and 3D-T2w MRI, were consistent to (0.41 ± 0.37) %. 4D-T2w MRI displayed tumour extent, structure and position more clearly than corresponding 4D-T1w MRI, especially when mobile tumour sites were adjacent to organs at risk.

*Conclusions*

A methodology to obtain 4D-T2w MRI that retrospectively applies the motion information from 4D-T1w MRI to 3D-T2w MRI was developed and verified. 4D-T2w MRI can assist clinicians in delineating mobile lesions that are difficult to define on 4D-T1w MRI, because of poor tumour-tissue contrast.

*Keywords*

4D MRI, motion model, motion vector field, MR-linac, radiotherapy treatment planning, 4D T2w MRI.

*Introduction*

Designing a radiotherapy treatment plan (RTP) for lung and abdominal cancer is challenging due to motion of the abdominal-thoracic region ^1^. An appropriate RTP should deliver the prescribed dose to the target and minimise dose to radiosensitive healthy structures. Unlike conventional free breathing scans where generic margins are added, four-dimensional (4D) images provide information on individual tumour motion, which can be used to generate a more personalised RTP ^2^.

Strategies have been proposed to manage abdominal-thoracic motion, such as breath-hold or gated treatment ^1^. Yet these approaches are sub-optimal, since they can be challenging for patients with primary lung malignancies or can lengthen treatment time. Motion information from image guided radiotherapy (IGRT) is used to improve treatment delivery ^3^.

Several imaging modalities can be employed for IGRT, such as orthogonal kV imaging, cone-beam computed tomography (CBCT) or, with the advent of hybrid MR-IGRT systems ^4-8^, magnetic resonance imaging (MRI). Compared to CBCT, MRI exhibits improved soft-tissue contrast, is a non-ionizing modality, and offers a range of clinically relevant contrasts ^9^. However, MRI is limited by intrinsic spatial uncertainty ^10^ and there is no direct relationship between electron density and the MRI signal, while a knowledge of electron density is required for dose calculations ^11^.

In this manuscript, 4D-MRI is defined as a time-series of 3-dimensional (3D) MRI image volumes, where each volume corresponds to a different time point in the respiratory cycle. In current clinical practice, 4D-MRI could be used to inform a RTP by providing additional information, such as improved soft-tissue contrast, absent in 4D-CT ^9^. In the near future, 4D-MRI might be acquired on hybrid MR-IGRT systems to provide information to support RTP adaptation with improved setup and beam-guidance ^5^. Thus, there is a great need for robust methods for generating 4D-MRI.

Previous work to produce 4D-MRI can be loosely split into dynamic slice-selective 2-dimensional (2D) and volumetric 3D acquisition schemes. 2D-MRI can be prospectively acquired at specific respiratory phases by triggering acquisition with the aid of a synchronised respiratory signal. A complete 4D-MRI volume is then constructed by continuously measuring slices at all required planes and respiratory phases ^12^. However triggering delays have been reported, where triggering of the current phase is ignored due to continuing measurement of the preceding phase, which can result in a long acquisition time ^13^.

Alternatively, 4D-MRI can be constructed retrospectively by sorting measured slices with the aid of a respiratory signal. Both external and internal respiratory signals have been utilised. Amongst others the centre of k-space (self-gating) ^14,15^, body area ^16^ and diaphragm position ^17-19^ have been employed as internal respiratory signals. External respiratory signals are typically acquired using a breathing belt ^20-23^, but clock drifts or poor respiratory correlation have been reported ^18,24^. Most commonly, the amplitude or phase of the respiratory signal has been applied to sort slices into bins of respiratory phase. Retrospective schemes suffer from data incompleteness artefacts, where a slice was not acquired in all respiratory phases, but can be mitigated by prolonged image acquisition ^18,20^.

4D-MRI constructed from aggregated and sorted 2D-MRI often exhibits: staircase artefacts, due to highly non-isotropic voxel sizes; low temporal resolution, because of a limited acquisition time and possible through-plane geometrical distortion, since scanner software typically only corrects in-plane distortion. This last point has been addressed by some authors who have reported application of an offline 3D distortion correction to 2D-MRI ^18^.

3D acquisition schemes do not have the limitations of 2D acquisition schemes and resulting 4D-MRI could be of higher quality. Yet translating methods used in 2D acquisition schemes ^12,13,16,18,20,25^ to 3D acquisition schemes is challenging, since dynamic 3D-MRI cannot typically be acquired with sufficient spatio-temporal resolution. However, good-quality 4D-MRI has been obtained using advanced offline-reconstructions of highly under-sampled data acquired using 3D non-Cartesian sequences ^15,26-29^.

Alternatively, 4D-MRI can be generated by applying a motion model to a reference volume ^30^. Blackall et al obtained 4D-MRI by applying a single-parameter motion model, with motion vector fields (MVF)s extracted from intraoperative ultrasound images, to static MRI ^31^. In likewise methods: McClelland et al developed a temporal-fitting motion model for CT, which was based on performing deformable image registration between reference and free breathing volumes ^32^; Marx et al generated 4D-CT by applying the motion information from 4D-MRI to 3D-CT ^33^. Recently, Stemkens et al proposed a motion model to generate real-time 4D-MRI, where a set of MVFs were obtained based on dynamic 2D-MRI and a reference volume ^34^.

To our knowledge, no method or motion model has been applied to generate 4D-T2w MRI from data measured with a 3D acquisition scheme. This might be because it is difficult to acquire dynamic 3D-T2w MRI, due to the long echo and repetition time required to achieve T2w contrast. Obtaining T2w MRI for lung RTP and guidance is important since T2w MRI is sensitive to both lung infiltrates and lesions with fluid content ^35^. Furthermore, T2w MRI enables an improved visualisation of both mobile organs at risk (OAR)s and tumour sites when compared to T1w MRI, for cases such as oesophageal cancer ^36^.

In this paper we: 1) introduce the motion vector field projection (MVFP) method, which provides a workflow to generate 4D-T2w MRI by applying the motion information from a 4D-T1w volume to a 3D-T2w volume; 2) verify calculated 4D-T2w MRI by comparing diaphragm positions, anatomical landmarks and volumetric image similarity in generated 4D-T2w MRI to 4D-T1w MRI; 3) discuss examples where 4D-T2w MRI more clearly shows tumour position, structure and extent when compared to 4D-T1w MRI.

*Materials and Methods*

*Data acquisition*

Ten patients with non-small cell lung cancer (6 female, 4 male, aged 63-86 years, 5 squamous cell carcinoma and 5 adenocarcinoma) were scanned with an axial 3D-T1w stack-of-stars spoiled gradient echo sequence in free breathing with golden angle spacing ^37,38^ and an axial 3D-T2w turbo spin echo sequence ^39^ with respiratory gating to exhalation at 1.5 T (MAGNETOM Aera, Siemens Healthcare, Erlangen, Germany). Axial orientation was chosen to facilitate delineation for the purpose of RTP. The T2w sequence was gated to exhalation using a liver dome navigator ^40^. The T1w sequence utilised a radial encoding scheme in the readout plane and a Cartesian slice encoding scheme. Each consecutive radial plane was obtained after rotating by the golden angle (θ ≈ 111.25**°**) ^38^.

A range of sequence parameters were employed, due to variation in patient habitus. Initially, a relatively high bandwidth (1085 Hz) was selected (patients 1 to 6), but was later found to be sub-optimal regarding image quality of reconstructed 4D-T1w MRI. After protocol optimisation, a lower bandwidth (630 Hz) was employed (patients 7 to 10), which resulted in an incremental improvement in image quality of 4D-T1w MRI and also enabled a smaller voxel size. Detailed acquisition parameters are listed in Table 1.

*Reconstruction of T1w data*

The acquired data were retrospectively reconstructed using the 4D joint motion-compensated high-dimensional total variation (4D joint MoCo-HDTV) algorithm ^15^. Before reconstruction, an adaptive gradient-delay compensation was applied to the raw data, so that artefacts associated with inaccuracies in the timing of gradients were reduced ^41^. Afterwards, the raw data were sorted into 20 overlapping respiratory phases based on the amplitude of the self-gating respiratory surrogate signal. The self-gating signal was extracted from the magnitude of the nine central k-space points on each radial spoke that passed through the k-space centre. Weightings of the HDTV operator were optimised by reducing temporal regularisation such that the images remained clear from under-sampling artefacts, whilst avoiding over-regularisation of true motion. Subsequently, an offline gradient non-linearity distortion correction was applied to each respiratory phase of the reconstructed 4D-T1w volume, using a spherical harmonics deconvolution method ^42,43^.

*Overview of the motion vector field projection method*

The motion vector field projection (MVFP) method generates 4D-T2w MRI by extracting motion information from 4D-T1w MRI and applying it, using a chain method, to 3D-T2w MRI. An overview of the MVFP method is displayed in Figure 1.

*Calculation and application of motion vector fields*

A one-dimensional (1D) signal describing image similarity was obtained by calculating the normalised mutual information (NMI) ^44^ (calculated in-plane and averaged over all slices) between the 3D-T2w volume and each respiratory phase j contained in the 4D-T1w volume; where j ∈ N (total number) respiratory phases. The respiratory phase i of the 3D-T2w volume was set equal to the respiratory phase j that corresponded to the signal maximum. In this way, a tie-phase was established between the 4D-T1w and 3D-T2w volumes.

MVFs between the respiratory phases i and j of the reconstructed volumes ($\boldsymbol{T}\boldsymbol{1}_{\boldsymbol{i}} ,\boldsymbol{T}\boldsymbol{1}_{\boldsymbol{j}}$) were calculated by deformable image registration. A b-spline GPU accelerated implementation of NiftyReg ^45,46^ was used to calculate all deformable image registrations.

A chain method, similar to that proposed by Boldea et al ^47^, was developed where the 3D-T1w volume at the n^th^ phase ${\boldsymbol{(T}\boldsymbol{1}}_{\boldsymbol{n}}^{\boldsymbol{'}}\boldsymbol{)}$ was obtained by sequentially applying a number of smaller deformations that are linked together at calculated chain-point phases, to the tie-phase$\left( \boldsymbol{T}\boldsymbol{1}_{\boldsymbol{i}} \right)$. The chain-method enables a balance between errors resulting from large deformations and concatenation of sequential deformations. An overview of the chain method can be found in Figure 2.

The chain-point phases were calculated based on fractions of the calculated image similarity signal range. This is possible because the image similarity signal indirectly corresponds to the deformation size between phases. The chain-point phases corresponding to inspiration were set equal to those of expiration, which is feasible because of the symmetry present in the respiratory pattern. A maximum of three deformations were chosen and phases closest to 33% and 67% of the signal range were set as chain-points.

The parameters of NiftyReg were optimised by comparing the estimated $(\boldsymbol{T}\boldsymbol{1}^{\boldsymbol{'}}\boldsymbol{)}$ and reconstructed $\left( \boldsymbol{T}\boldsymbol{1} \right)$ 4D-T1w volumes. The root mean square error (RMSE), dice coefficient (averaged over all respiratory phases) and visual image quality were analysed as metrics. A NMI cost function with: number of levels performed = 3, control point spacing = 3 mm, bending energy weighting = 0, Jacobian penalty weighting = 0 and maximum iterations = 500, was best able to reproduce the reconstructed 4D-T1w volume (Dice > 0.965, RMSE < 5%, good qualitative agreement).

The 3D-T2w volume was registered and then interpolated to the matrix size of the 4D-T1w tie-phase. Calculated 4D-T2w MRI is then obtained by applying the calculated MVFs, employing the chain method, to the registered and interpolated 3D-T2w volume.

All calculations were undertaken on an Intel Xeon E5-1660 processor with 8 cores at 3 GHz and 64 GB of memory.

*Verification of the 4D-T2w volumes*

The calculated 4D-T2w volumes were verified against their corresponding 4D-T1w volumes. Using MATLAB (The MathWorks, Natick, MA), a semi-automated edge-detection method was developed and employed to verify the diaphragm positions, a radiation oncologist manually delineated control points for comparison of anatomical positions and the NMI was calculated to assess volumetric image similarity. For each metric, the differences between 4D-T1w and 4D-T2w MRI were compared to those between 4D-T1w and 3D-T2w MRI.

In the edge-detection method, the user manually places a rectangular 2D region of interest (ROI) over the right hemi-diaphragm surface, orientated along the superior-inferior (SI) direction, on a coronal or sagittal slice. Along the SI dimension the ROI should be sufficiently large to encompass expected respiratory motion (approximately 2-3cm) and narrow in the medial-lateral dimension such that the bounded diaphragm surface is approximately flat. For consistency, a ROI was placed on the coronal slice that exhibited both the aortic arch and the descending aorta.

Intensity was measured along all SI lines within the 2D ROI. For each SI line the diaphragm position was calculated by fitting a Gaussian to the derivative of the measured line intensity. To increase robustness a ramp function was employed. Outliers in diaphragm position across all SI lines were removed based on the inter-quartile range of the detected diaphragm positions. The diaphragm position was chosen as the mean of the remaining diaphragm positions within the 2D ROI and the standard deviation corresponds to the diaphragm width.

Using an in-house developed delineation toolkit, the radiation oncologist (6 years experience) identified five anatomical control points (one static and four mobile) in even respiratory phases in both 4D-T1w and 4D-T2w MRI:

1. Posterior spinal canal at the superior aspect of T4.
2. Inferior point of the carina.
3. Bifurcation of the right middle and lower lobe bronchus.
4. Bifurcation of the left main bronchus and left upper lobe bronchus.
5. Right costophrenic angle.

The right costophrenic angle was delineated on coronal images, with the coronal slice chosen to correspond with the level of the bifurcation of the right upper and middle lobe bronchus. The delineation toolkit offered coronal, sagittal and axial views and delineation could be performed on either of them. Figure 3 shows an example of control point delineation for patient 8. These particular control points were chosen because they are visible in both 4D-T1w and 4D-T2w MRI, as well as being reproducible across patient sets. To assess spatial coherence between landmarks, Euclidean distances were calculated between pairs of delineated points in both 4D-T1w and 4D-T2w MRI.

NMI ^44^ was calculated between each corresponding respiratory phase of the 4D-T1w and 4D-T2w volumes. The result was compared to the NMI measured between each respiratory phase of the 4D-T1w volume and the 3D-T2w volume.

If 4D-T1w and 4D-T2w MRI are commensurate in respiratory phase and geometrical position, then the NMI for all respiratory phases should be similar to the NMI assessed between the tie-phase of the 4D-T1w volume and the 3D-T2w volume.

*Results*

4D-T2w MRI was calculated for ten patients with primary lung malignancies. 4D-T1w MRI reconstruction, using a non-optimised prototype implementation, took between 9-12 hours for 20 respiratory phases and the chain-method took between 25-30 minutes. The mean period of the respiratory cycle averaged over patients was (4.1 ± 0.95) s. Figure 4 shows an example reconstructed 4D-T1w volume and a calculated 4D-T2w volume at respiratory phases corresponding to exhalation, mid-cycle and inhalation. The movie in Supplemental Digital Content 1, <http://links.lww.com/RLI/A316>, displays a similar example, but with all respiratory phases. For all patients, 4D-T2w MRI exhibited qualitatively similar respiratory motion to corresponding 4D-T1w MRI.

*Verification of 4D-T2w MRI*

Mean diaphragm surface positions were calculated using the edge-detection method for all patients on both T1w and T2w images. Figure 5a displays an example of the diaphragm surface displacement for patient 5. Figure 5b shows the range of diaphragmatic displacement of 4D-T1w MRI, as calculated by the edge-detection method. Figure 5c shows the spread of the displacement between diaphragm positions on both T1w and T2w images across all respiratory phases. Median diaphragm positions were consistent to less than one slice thickness (3.3 mm) for all patients, except patient 7, which exhibited median displacements within 6.6 mm. For all patients, T1w and T2w MRI were less consistent at inhalation than at exhalation. The Pearson correlation coefficient was calculated between the median diaphragm differences of 4D-T1w and 4D-T2w MRI, and the range of diaphragmatic displacements in 4D-T1w MRI. No significant correlation (r = -0.19, p = 0.60) was observed.

There was a reduction in the mean and standard deviation, averaged over all patients, of the interquartile range of the differences in diaphragm positions over all respiratory phases, between 4D-T1w and 4D-T2w MRI (1.11 ± 0.81) mm and between 4D-T1w and 3D-T2w MRI (9.83 ± 3.95) mm.

A radiation oncologist manually delineated five anatomical landmarks on both 4D-T1w and 4D-T2w MRI, and the Euclidean distances between them were calculated. Results are shown in Figure 6.

Euclidean distances between all corresponding anatomical landmarks were within or better than 7.6 mm (Euclidean distance of two voxels) and less than 3.8 mm (Euclidean distance of one voxel) for 355 out of 470 delineated pairs of anatomical control points. Three anatomical landmarks were excluded from delineation: for patient 2, the posterior spinal canal at the superior aspect of T4 was removed because of poor visibility on T2w MRI; for patient 6, the bifurcation of the left main bronchus and left upper lobe bronchus was omitted due to partial collapse of the left upper lobe; for patient 4, the right costophrenic angle was outside the acquired field of view.

There was a reduction in the mean and standard deviation, averaged over patients, of the interquartile range, calculated over respiratory phases, of the Euclidean distances for the mobile ROIs (ROIs: 2-5) between 4D-T1w and 4D-T2w MRI: (0.80 ± 0.35, 0.85 ± 0.39, 0.78 ± 0.28,1.62 ± 0.70) mm and between 4D-T1w and 3D-T2w MRI: (1.23 ± 0.48, 2.02 ± 0.98, 1.19 ± 0.94, 5.16 ± 2.08) mm.

The NMI was calculated between corresponding respiratory phases of 4D-T1w and 4D-T2w MRI, and was compared to the NMI of 4D-T1w and 3D-T2w MRI. Comparisons were made in relation to the NMI calculated between the tie-phase of 4D-T1w and 3D-T2w MRI. Figure 7 shows an example comparison, for patient 10. Percentage differences in NMI (mean and standard deviation, calculated over all respiratory phases and patients) of 4D-T1w and 4D-T2w MRI were (0.41 ± 0.37) % and between 4D-T1w and 3D-T2w MRI were (-1.82 ± 1.76) %.

*Image artefacts*

The dominant image artefact observed after application of the MVFP method was associated with inaccuracies in deformable image registration, which led to a reduction in the quality of 4D-T2w MRI for all patients and was found to increase in magnitude with deformation size. An example is shown in Figure 8.

Artefacts that were already present in 3D-T2w MRI were propagated into 4D-T2w MRI. For all patients minor ghosting was displayed and for two patients intensity inhomogeneity was apparent.

*Clinical evaluation*

The radiation oncologist reported improved tumour definition in 4D-T2w MRI when compared to 4D-T1w MRI. Furthermore, important clinical information is displayed. In the case presented in Figure 4, T2w MRI better highlights extent and position of reactive pleural effusion and the lymphadenopathy than T1w MRI.

In some situations, 4D-T2w MRI was particularly advantageous when compared to 4D-T1w MRI, for instance when mobile tumour beds were attached to or adjacent to OARs, as demonstrated in Figure 9 and in the movie provided as Supplemental Digital Content 2, http://links.lww.com/RLI/A317. In the T1w images, the tumour-tissue contrast is low and it is challenging to delineate the tumour. However, the tumour extent and structure is clearly visible in T2w MRI. Furthermore, the anterior part of the tumour site is sliding non-rigidly against the chest-wall and the extent of attachment and sliding motion is more easily visualised in T2w MRI than T1w MRI.

*Discussion*

*Pulse sequences*

A sequence with a radial trajectory and golden angle spacing was selected to acquire the T1w data, because of advantages in image quality, such as incoherent aliasing and insensitivity to motion ^37^ and because a self-gating signal could be obtained through frequent measurements of the k-space centre ^48^. Alternative non-Cartesian trajectories could be used ^26^, but a stack-of-stars approach is more efficient during reconstruction, as a Fourier transform can be applied along the slice direction ^37^.

We employed an incremental improvement during the study in reducing the readout bandwidth of the 4D-T1w MRI acquisition. However, there was no significant difference in median diaphragm displacements on T1w and T2w MRI between patients 1 to 6 and patients 7 to 10 (unpaired two-tailed t-test, significance level α = 0.05, p = 0.34). Therefore a group analysis was justified.

The chosen T2w sequence employs a variable flip angle distribution to extend the echo train and speed up acquisition, enabling volumetric T2w MRI to be acquired in a clinically acceptable time period (5-9 min). The application of a 3D acquisition is preferable to 2D slice-selective excitation sequences, commonly used in alternative 4D-MRI methods ^12,13,16,18,20,25^, since the resulting MRI provides improved SNR. This property enables thinner slices and consequently a reduction in stair-case artefacts due to highly non-isotropic voxel sizes. Yet, in ^13^ 4D-T2w MRI was calculated with a similar slice thickness of 3 mm, but a comparatively smaller field of view along the slice-direction was acquired. Furthermore, employing 3D sequences might improve image quality, for delineation purposes, by reducing the influence of a complex magnetization history (impact of in-flow, slice-selection pulses, excitation frequency). Primarily, 3D sequences offer improved geometrical fidelity when using 3D distortion correction, which on commercially available systems is often not available for 2D sequences, but nonetheless is essential for RTP.

*Image reconstruction*

The 4D joint MoCo-HDTV reconstruction was chosen, since it not only enables high undersampling factors by employing 100% of the raw data for reconstruction of each respiratory phase, but also results in good image quality with comparatively low streaking artefacts and high sharpness ^15^. There are alternative reconstructions which utilise similar methods, but are not yet readily available for the whole thorax ^49^.

*Artefacts apparent using the motion vector field projection method*

The MVFP method has limitations. The deformation artefact causes the diaphragm surface to be discontinuous and predominantly occurs at inhalation. A similar artefact has been reported when using NiftyReg with lung 4D-CT data ^50^. In our case, the artefact is present in the estimated 4D-T1w volume $(\boldsymbol{T}\boldsymbol{1}^{\boldsymbol{'}}\boldsymbol{)}$, but not in the reconstructed 4D-T1w volume $(\boldsymbol{T}\boldsymbol{1)}$, and therefore is due to errors accumulated during application of the chain-method. The extent of the artefact can be loosely quantified as the interquartile range of the difference in diaphragm positions between 4D-T1w and 4D-T2w MRI, the mean (calculated over all patients) of which is 1.11 mm (less than 0.5 voxel); demonstrating that the artefact has only a minor impact on diaphragm continuity of 4D-T2w MRI.

*Verification*

4D-T2w MRI was verified with respect to 4D-T1w MRI. Another option might have been to compare the inhalation respiratory phase of 4D-T2w MRI to T2w MRI acquired in breath-hold. However this approach was not pursued since inhalation in breath-hold can be deeper than in free-breathing ^30,51^.

*Diaphragm positions*

The median diaphragm positions were consistent to less than 6.6 mm (2 voxels) for all ten patients and less than 3.3 mm (1 voxel) for nine of ten patients. Furthermore, the mean of the interquartile range of observed differences between 4D-T1w and 4D-T2w MRI was ≈ 8.7 mm smaller than between 4D-T1w and 3D-T2w MRI, demonstrating that calculated 4D-T2w MRI not only contained similar motion information to 4D-T1w MRI, but was also spatially coherent.

The observed differences are partly due to a mismatch in respiratory phase between 4D-T1w and 3D-T2w MRI, as for all patients, comparison of the diaphragm positions (using the edge-detection method) in 4D-T1w and 3D-T2w MRI indicated that no exact match could be found. This might be solved by non-rigidly warping the 3D-T2w volume to the closest matching phase of the 4D-T1w volume. One hypothesis was that the differences depended on the magnitude of the diaphragm displacement during the respiratory cycle, but no significant correlation was found.

The larger differences observed for patient 7 might result from a collapse of the middle lobe of the right lung, as can be seen in the movie of Supplemental Digital Content 1, http://links.lww.com/RLI/A316.

*Anatomical control points*

4D-T2w MRI was anatomically similar to 4D-T1w MRI since all Euclidean distances, calculated between corresponding control points, were consistent to less than 7.6 mm (Euclidean distance of two voxels) and Euclidean distances of 355 out of 470 pairs of control points agreed to less than 3.8 mm (Euclidean distance of one voxel). Applying the MVFP method led to improved similarity of T1w and T2w MRI as the mean interquartile range of Euclidean distances between mobile ROIs of 4D-T1w and 4D-T2w MRI were smaller than those between 4D-T1w and 3D-T2w MRI by ≈ 1.39 mm.

Verification by manual delineation is limited, since it is a subjective process such that part of the presented differences, between pairs of anatomical control points, could be attributed to user dependence. Multiple observers might increase the accuracy of determined anatomical landmark positions.

A single observer approach was undertaken to reflect likely clinical practice, which could involve manual delineation on several respiratory phases.

*Volumetric image similarity*

A high volumetric image similarity was observed between 4D-T1w and 4D-T2w MRI, as NMI, relative to the tie-phase of 4D-T1w and 3D-T2w MRI, was coherent within (0.41 ± 0.37) %. No dependence on respiration of the NMI calculated between corresponding respiratory phases of 4D-T1w and 4D-T2w MRI is apparent in Figure 7. 4D-T2w MRI is thus commensurate to 4D-T1w MRI in spatio-temporal location.

A drawback of this approach is that the NMI metric is not only sensitive to relevant spatio-temporal information, but also to image artefacts and noise. This metric was chosen because it can handle analysis between different image contrasts ^44^.

*Application*

As a proof-of-principle, high quality geometrically accurate 4D-T2w images were calculated and could assist clinicians in obtaining RTPs for anatomical regions affected by respiratory motion by improving tumour definition compared to 4D-T1w or 4D-CT images. For instance, calculated MVFs might be applied to the 3D-T2w volume to generate mid-vent T2w MRI, which could be used alongside mid-vent CT ^2^, to aid delineation of malignant tissue. The current workflow for radiotherapy planning and delineation includes a free breathing ^18^F-fluorodexoyglucose position emission tomography (PET) scan. 4D PET imaging has been proposed, but further clinical validation is required ^52^ and unlike the presented 4D-T2w MRI, has limited spatial resolution (5-7 mm) ^53,54^.

The improved tumour-tissue contrast exhibited by 4D-T2w MRI could be particularly beneficial when moving tumour sites are adjacent to OARs, since it can often be challenging to delineate such sites on 4D-T1w MRI or 4D-CT ^36,55^. Additionally, 4D-T2w MRI could improve the reliability and specificity of assessment of chest wall invasion when compared to 4D-T1w MRI and 4D-CT ^56^.

4D-T2w MRI could be used as part of an MR only workflow for hybrid MR-IGRT systems ^4-8^. For instance, high quality 4D-T2w MRI could act as a reference volume for both retrospective evaluation of the delivered treatment and the generation of real-time 4D-T2w MRI for beam-on guidance and planning, in methods as proposed by Stemkens et al ^34^.

The MVFP method is not limited to 4D-T2w MRI and could be applied to simulate 4D-MRI displaying any required contrast. For specific contrasts, such as diffusion weighted MRI or ultrashort echo time imaging, it is not possible to use a stack-of-stars k-space sampling ^57^, making it more difficult to apply state of the art 4D reconstruction methods. In this case, the MVFP method could act as a solution to generate high quality 4D-MRI.

*Conclusion*

4D-T2w MRI was calculated retrospectively by applying the motion information from a 4D-T1w volume to a static 3D-T2w volume. Good quality geometrically accurate 4D-T2w volumes were obtained, providing high spatio-temporal resolution. 4D-T2w MRI may assist clinicians in delineating lesions within volumes affected by respiratory motion that are challenging to outline on a 4D-T1w volume, making it a promising candidate for applications in radiotherapy, particularly with hybrid MR-IGRT systems in mind.

References

1. Keall PJ, Mageras GS, Balter JM, et al. The management of respiratory motion in radiation oncology report of AAPM Task Group 76a). *Med Phys*. 2006;33:3874-3900.

2. Wolthaus JW, Sonke J-J, van Herk M, et al. Comparison of different strategies to use four-dimensional computed tomography in treatment planning for lung cancer patients. *Int J Radiat Oncol*. 2008;70:1229-1238.

3. Sonke JJ, Zijp L, Remeijer P, et al. Respiratory correlated cone beam CT. *Med Phys*. 2005;32:1176-1186.

4. Raaymakers B, Lagendijk J, Overweg J, et al. Integrating a 1.5 T MRI scanner with a 6 MV accelerator: proof of concept. *Phys Med Biol*. 2009;54:N229.

5. Lagendijk JJ, Raaymakers BW, Van den Berg CA, et al. MR guidance in radiotherapy. *Phys Med Biol*. 2014;59:R349.

6. Fallone BG. The rotating biplanar linac–magnetic resonance imaging system. *Semin Radiat Oncol*. 2014;24:200-202.

7. Mutic S, Dempsey JF. The ViewRay system: magnetic resonance–guided and controlled radiotherapy. *Semin Radiat Oncol*. 2014;24:196-199.

8. Thwaites D, Keall P, Holloway L, et al. Observations on MR-LINAC systems and rationale for MR-Linac use: The australian MR-Linac project as an example. *Phys Medica*. 2014;30:e25.

9. Schmidt MA, Payne GS. Radiotherapy planning using MRI. *Phys Med Biol*. 2015;60:R323.

10. Weygand J, Fuller CD, Ibbott GS, et al. Spatial Precision in Magnetic Resonance Imaging-Guided Radiotherapy: The Role of Geometric Distortion. *Int J Radiat Oncol*. 2016;95:1304-1316.

11. Hofmann M, Pichler B, Schölkopf B, et al. Towards quantitative PET/MRI: a review of MR-based attenuation correction techniques. *Eur J Nucl Med Mol I*. 2009;36:93-104.

12. Hu Y, Caruthers SD, Low DA, et al. Respiratory amplitude guided 4-dimensional magnetic resonance imaging. *Int J Radiat Oncol*. 2013;86:198-204.

13. Du D, Caruthers SD, Glide-Hurst C, et al. High-quality t2-weighted 4-dimensional magnetic resonance imaging for radiation therapy applications. *Int J Radiat Oncol*. 2015;92:430-437.

14. Larson AC, Kellman P, Arai A, et al. Preliminary investigation of respiratory self‐gating for free‐breathing segmented cine MRI. *Magn Reson Med*. 2005;53:159-168.

15. Rank CM, Heußer T, Buzan MT, et al. 4D respiratory motion‐compensated image reconstruction of free‐breathing radial MR data with very high undersampling. *Magn Reson Med*. 2017;77:1170-1183.

16. Cai J, Chang Z, Wang Z, et al. Four-dimensional magnetic resonance imaging (4D-MRI) using image-based respiratory surrogate: A feasibility study. *Med Phys*. 2011;38:6384-6394.

17. King AP, Buerger C, Tsoumpas C, et al. Thoracic respiratory motion estimation from MRI using a statistical model and a 2-D image navigator. *Med Image Anal.* 2012;16:252-264.

18. Tryggestad E, Flammang A, Han-Oh S, et al. Respiration-based sorting of dynamic MRI to derive representative 4D-MRI for radiotherapy planning. *Med Phys*. 2013;40:051909.

19. Savill F, Schaeffter T, King AP. Assessment of input signal positioning for cardiac respiratory motion models during different breathing patterns. *IEEE International Symposium on Biomedical Imaging: From Nano to Macro*. 2011:1698-1701.

20. Liu Y, Yin F-F, Czito BG, et al. T2-weighted four dimensional magnetic resonance imaging with result-driven phase sorting. *Med Phys*. 2015;42:4460-4471.

21. Vedam S, Keall P, Kini V, et al. Acquiring a four-dimensional computed tomography dataset using an external respiratory signal. *Phys Med Biol*. 2002;48:45-62.

22. Hui C, Wen Z, Stemkens B, et al. 4D MR imaging using robust internal r espiratory signal. *Phys Med Biol*. 2016;61:3472-3487.

23. Li G, Wei J, Olek D, et al. Direct comparison of respiration-correlated four-dimensional magnetic resonance imaging (4DMRI) reconstructed based on concurrent internal navigator and external bellows. *Int J Radiat Oncol*. 2016;97:596-605.

24. Koch N, Liu HH, Starkschall G, et al. Evaluation of internal lung motion for respiratory-gated radiotherapy using MRI: Part I—correlating internal lung motion with skin fiducial motion. *Int J Radiat Oncol*. 2004;60:1459-1472.

25. Liu Y, Yin F-F, Chen N-k, et al. Four dimensional magnetic resonance imaging with retrospective k-space reordering: A feasibility study. *Med Phys*. 2015;42:534-541.

26. Deng Z, Pang J, Yang W, et al. Four‐dimensional MRI using three‐dimensional radial sampling with respiratory self‐gating to characterize temporal phase‐resolved respiratory motion in the abdomen. *Magn Reson Med*. 2015;75:1574-1585.

27. Feng L, Axel L, Chandarana H, et al. XD‐GRASP: Golden‐angle radial MRI with reconstruction of extra motion‐state dimensions using compressed sensing. *Magn Reson Med*. 2016;75:775-788.

28. Feng L, Grimm R, Block KT, et al. Golden‐angle radial sparse parallel MRI: Combination of compressed sensing, parallel imaging, and golden‐angle radial sampling for fast and flexible dynamic volumetric MRI. *Magn Reson* *Med*. 2014;72:707-717.

29. Mickevicius NJ, Paulson E. Investigation of undersampling and reconstruction algorithm dependence on respiratory correlated 4D-MRI for online MR-guided radiation therapy. *Phys Med Biol*. 2016. [Epub ahead of print].

30. McClelland JR, Hawkes DJ, Schaeffter T, et al. Respiratory motion models: a review. *Med Image Anal*. 2013;17:19-42.

31. Blackall JM, Penney GP, King AP, et al. Alignment of sparse freehand 3-D ultrasound with preoperative images of the liver using models of respiratory motion and deformation. *IEEE T Med Imaging*. 2005;24:1405-1416.

32. McClelland JR, Blackall JM, Tarte S, et al. A continuous 4D motion model from multiple respiratory cycles for use in lung radiotherapy. *Med Phys*. 2006;33:3348-3358.

33. Marx M, Ehrhardt J, Werner R, et al. Simulation of spatiotemporal CT data sets using a 4D MRI-based lung motion model. *Int J Comput Ass Rad*. 2014;9:401-409.

34. Stemkens B, Tijssen RH, de Senneville BD, et al. Image-driven, model-based 3D abdominal motion estimation for MR-guided radiotherapy. *Phys Med Biol*. 2016;61:5335-5355.

35. Biederer J, Beer M, Hirsch W, et al. MRI of the lung (2/3). Why… when… how? *Insights Imaging*. 2012;3:355-371.

36. Riddell AM, Hillier J, Brown G, et al. Potential of surface-coil MRI for staging of esophageal cancer. *Am J Roentgenol*. 2006;187:1280-1287.

37. Block KT, Chandarana H, Milla S, et al. Towards routine clinical use of radial stack-of-stars 3d gradient-echo sequences for reducing motion sensitivity. *J Korean Phys Soc*. 2014;18:87-106.

38. Winkelmann S, Schaeffter T, Koehler T, et al. An optimal radial profile order based on the Golden Ratio for time-resolved MRI. *IEEE T Med Imaging*. 2007;26:68-76.

39. Lichy MP, Wietek BM, Mugler III JP, et al. Magnetic resonance imaging of the body trunk using a single-slab, 3-dimensional, T2-weighted turbo-spin-echo sequence with high sampling efficiency (SPACE) for high spatial resolution imaging: initial clinical experiences. *Invest Radiol*. 2005;40:754-760.

40. Danias PG, McConnell MV, Khasgiwala VC, et al. Prospective navigator correction of image position for coronary MR angiography. *Radiology*. 1997;203:733-736.

41. Block K, Uecker M. Simple method for adaptive gradient-delay compensation in radial MRI. *ISMRM 19th Annual Meeting & Exhibition*. 2011:2816.

42. Doran SJ, Charles-Edwards L, Reinsberg SA, et al. A complete distortion correction for MR images: I. Gradient warp correction. *Phys Med Biol*. 2005;50:1343.

43. Janke A, Zhao H, Cowin GJ, et al. Use of spherical harmonic deconvolution methods to compensate for nonlinear gradient effects on MRI images. *Magn Reson Med*. 2004;52:115-122.

44. Pluim JP, Maintz JA, Viergever MA. Mutual-information-based registration of medical images: a survey. *IEEE T Med Imaging.* 2003;22:986-1004.

45. Modat M, Ridgway GR, Taylor ZA, et al. Fast free-form deformation using graphics processing units. *Comput Meth Prog Bio*. 2010;98:278-284.

46. Rueckert D, Sonoda LI, Hayes C, et al. Nonrigid registration using free-form deformations: application to breast MR images. *IEEE T Med Imaging*. 1999;18:712-721.

47. Boldea V, Sharp GC, Jiang SB, et al. 4D-CT lung motion estimation with deformable registration: quantification of motion nonlinearity and hysteresis. *Med Phys*. 2008;35:1008-1018.

48. Buehrer M, Curcic J, Boesiger P, et al. Prospective self‐gating for simultaneous compensation of cardiac and respiratory motion. *Magn Reson Med*. 2008;60:683-690.

49. Aitken AP, Henningsson M, Botnar RM, et al. 100% Efficient three‐dimensional coronary MR angiography with two‐dimensional beat‐to‐beat translational and bin‐to‐bin affine motion correction. *Magn Reson Med*. 2015;74:756-764.

50. Modat M, McClelland J, Ourselin S. Lung registration using the NiftyReg package. *Medical Image Analysis for the Clinic-A Grand Challenge*. 2010;2010:33-42.

51. Blackall J, Ahmad S, Miquel M, et al. MRI-based measurements of respiratory motion variability and assessment of imaging strategies for radiotherapy planning. *Phys Med Biol*. 2006;51:4147-4169.

52. Konert T, Vogel W, MacManus MP, et al. PET/CT imaging for target volume delineation in curative intent radiotherapy of non-small cell lung cancer: IAEA consensus report 2014. *Radiother Oncol*. 2015;116:27-34.

53. Kumar S, Liney G, Rai R, et al. Magnetic resonance imaging in lung: a review of its potential for radiotherapy. *Brit J Radiol*. 2016;89:20150431.

54. Hanna G, de Koste JvS, Dahele M, et al. Defining Target Volumes for Stereotactic Ablative Radiotherapy of Early-stage Lung Tumours: A Comparison of Three-dimensional 18 F-fluorodeoxyglucose Positron Emission Tomography and Four-dimensional Computed Tomography. *Clin Oncol*. 2012;24:e71-e80.

55. Brown G, Daniels I, Richardson C, et al. Techniques and trouble-shooting in high spatial resolution thin slice MRI for rectal cancer. *Brit J Radiol*. 2014;78:245-251.

56. Khalil A, Majlath M, Gounant V, et al. Contribution of magnetic resonance imaging in lung cancer imaging. *Diagn Interv Imaging*. 2016;97:991-1002.

57. Triphan SM, Jobst BJ, Breuer FA, et al. Echo time dependence of observed T1 in the human lung. *J Magn Reson Imaging*. 2015;42:610-616.

Table Legends

Table 1: Acquisition parameters of the T1w and T2w sequences. Patient habitus determined acquisition parameters. Using a lower bandwidth of 630 Hz for the T1w sequence (patients 7 to 10) resulted in slightly improved results of the joint MoCo-HDTV reconstruction. Fat suppression was used in 8 out of 10 cases for the T2w sequence.

Figure Legends

Figure 1): A visual overview of the motion vector field projection method. Note that the green, red and yellow arrows in step 4 display the motion vector fields corresponding to the shown axial, coronal and sagittal planes. Dashed white lines aid visualisation of the diaphragm position on T1w and T2w MRI.

Figure 2: a) Displays the image similarity signal with the tie-point phase (red square) and chain-point phases (blue circles). The values (horizontal solid lines) of the fractions of the signal range, used to calculate the chain-point phases, are shown. b) The volumes corresponding to the chain-point phases are determined by sequentially warping the tie-phase with the required motion vector fields (MVF)s. The chain-point phases are used as a starting point to generate any arbitrary phase. For instance, the m^th^ phase is calculated with two deformations (Tie-phase 🡪 Chain-point 1; Chain-point 1 🡪 m^th^ phase) and the n^th^ phase requires three deformations (Tie-phase 🡪 Chain-point 1; Chain-point 1 🡪 Chain-point 2; Chain-point 2 🡪 n^th^ phase).

Figure 3: An example of control point delineation for patient 8 on a snapshot image of 4D-T1w MRI. The control points (red circles) are contained within dashed red boxes to assist visualisation. The presented windowing scheme was optimised by the radiation oncologist for viewing of the anatomical landmark and not for surrounding anatomy.

Figure 4: Example T1w and T2w coronal, sagittal and axial views at exhalation, mid-cycle and inhalation respiratory phases for patient 7. This patient was diagnosed with T1aN2 primary lung squamous cell carcinoma and had a small benign reactive pleural effusion. The dashed white lines aid visualisation of the diaphragm surface position. The respiratory pattern of T1w MRI is preserved in T2w MRI. The tumour site, lymphadenopathy and reactive pleural effusion are shown more clearly in T2w MRI than T1w MRI.

Figure 5: a) Example diaphragm surface positions, calculated using the edge-detection method, of the 4D-T1w and 4D-T2w volumes for patient 5. Error bars correspond to the standard deviation across SI lines and represent the width of the diaphragm surface. b) The ranges of diaphragmatic displacements in 4D-T1w MRI calculated using the edge-detection method. Errors bars show the uncertainty in the range. c) Boxplot of the spread of the difference in diaphragm surface positions, between the 4D-T1w and 4D-T2w volumes, as calculated by the edge-detection method, for the ten patients.

Figure 6: Boxplot of spread of Euclidean distances, for all even respiratory phases, between pairs of anatomical landmarks that were delineated in both 4D-T1w and 4D-T2w MRI. All Euclidean distances agreed to less than 7.6 mm (Euclidean distance of two voxels) and 355 out of 470 pairs of anatomical landmarks were consistent to less than 3.8 mm (Euclidean distance of one voxel).

Figure 7: Shows an example comparison for patient 10, where results are relative to the normalised mutual information (NMI) calculated between the tie-phase of 4D-T1w and 3D-T2w MRI. In this case, the tie-phase is respiratory phase 1. NMI between 4D-T1w and 3D-T2w MRI (black solid line) indirectly corresponds to a respiratory signal. This pattern is not observed when examining NMI between 4D-T1w and 4D-T2w MRI (blue dashed curve).

Figure 8: An example of the deformation artefact for patient 7. In the T2w snapshots, a deformation of the right hemi-diaphragm boundary is seen at mid-cycle and a comparatively large deformation at inhalation (dashed circle). The deformation is worse at inhalation than at mid-cycle and is not displayed in the corresponding T1w snapshots.

Figure 9: Snapshots of 4D-T1w and 4D-T2w MRI at the exhalation and inhalation respiratory phases for patient 3, who was diagnosed with T4N2 adenocarcinoma. The tumour is radically treatable, but is embedded around the heart and oesophagus. Unlike T1w MRI, T2w MRI displays a high tumour-tissue contrast enabling tumour position and structure to be clearly delineated. Sliding motion of the tumour site against the chest wall is displayed in the sagittal plane and is more clearly presented in T2w than T1w MRI. Red arrows point to the tumour site and dashed white lines aid assessment of both superior-inferior diaphragm and sliding motion.

List of Supplemental Digital Content

Supplemental_Digital_Content_1.mp4

Supplemental_Digital_Content_2.mp4
